# Elucidating Tumorigenesis Mechanisms and Assessing Immunotherapeutic Efficacy in Patient-Derived Medulloblastoma Organoid Models

**DOI:** 10.7150/ijbs.116040

**Published:** 2026-04-23

**Authors:** Jiting Zhang, Min Wang, Huanwen Rui, Cen Wang, Guanghao Luo, Zhiyuan Niu, Wei Shi, Junwei Zeng, Ping Xue, Xueyao Shi, Bing Yan, Wenyan Ren, Hao Li, Xinhua Lin

**Affiliations:** 1 State Key Laboratory of Genetics and Development of Complex Phenotypes, School of Life Sciences, Greater Bay Area Institute of Precision Medicine (Guangzhou), Zhongshan Hospital, Fudan University, Shanghai, China.; 2 Human Phenome Institute, Fudan University, Shanghai, China.; 3 Department of Neurosurgery, Children's Hospital of Fudan University, National Children's Medical Center, Shanghai, China.; 4 Liangzhu Laboratory, Zhejiang University, Hangzhou, China.; 5 State Key Laboratory of Reproductive Regulation and Breeding of Grassland Livestock, Institute of Biomedical Sciences, School of Life Sciences, Inner Mongolia University, Hohhot, China.; 6 Jiangsu Key Laboratory of Drug Discovery and Translational Research for Brain Diseases, Cambridge-Suda Genomic Resource Center, Suzhou Medical College of Soochow University, Suzhou, China.

**Keywords:** medulloblastoma, organoids, single-cell sequencing, tumorigenesis, immunotherapy

## Abstract

Medulloblastoma is one of the most common malignant pediatric brain tumors. There remain significant challenges in investigating oncogenic mechanisms and evaluating therapeutic efficacy due to the limited available models that accurately reflect tumor heterogeneity. To overcome this limitation, we established 10 patient-derived medulloblastoma organoids (MBOs) that retain the histological characteristics, and cellular diversity of the original tumors. These MBOs demonstrate strong infiltration capabilities, both *in vitro* through co-culture with human embryonic stem cell-derived cerebral organoids and *in vivo* following orthotopic or subcutaneous transplantation, establishing a potential platform for investigating interactions within the tumor microenvironment. Using integrated RNA sequencing, whole-exome sequencing, and DNA methylation profiling, we demonstrated that MBOs faithfully preserve the transcriptional, genomic, and epigenetic landscapes of their parental tumors. Single-cell transcriptomic analysis revealed conserved cellular subpopulation between MBOs and primary tumors. Our findings suggest that photoreceptor-related pathways may play an unprecedented role in the pathogenesis of Group 4 medulloblastoma and may be associated with interactions within the tumor microenvironment. Furthermore, we developed a prognostic nomogram based on IMPG2, BNC2, PAPPA2, ITGBL1and UNC13C expression levels in tumor cells to predict survival outcomes. Notably, tumor-infiltrating lymphocytes (TILs) expanded from patient specimens exhibited significant cytotoxic activity against autologous MBOs co-cultured *in vitro* and effectively suppressed the growth of subcutaneous MBO xenografts *in vivo*. These findings demonstrate the potential of TIL-based immunotherapy for medulloblastoma treatment. Collectively, our MBO system faithfully recapitulates critical tumor characteristics and serves as a valuable platform for investigating tumorigenic mechanisms and assessing therapeutic responses. This study not only promotes fundamental biological research but also accelerates clinical translation in medulloblastoma.

## 1. Introduction

Medulloblastoma (MB), the most prevalent childhood primary malignancy of the central nervous system, accounts for approximately 25% of pediatric intracranial neoplasms 1. Medulloblastoma is classified as a germinal matrix epithelial tumor of the cerebellum and is divided into four molecular subgroups: WNT, SHH, Group 3, and Group 4^2^. These molecular subtypes originate from distinct progenitor cell populations through spatiotemporally restricted transformation processes. WNT MBs arise from progenitor cells in the extracerebellar lower rhombic lip, whereas SHH MBs originate from cerebellar granule neuron progenitors (GNPs)^3^. In contrast, the cellular origins of Group 3 and Group 4 MB are less understood due to their more complex subtypes. Recent studies have demonstrated that Group 3 and Group 4 MBs arise from cells in the subventricular zone of the rhombic lip (RL^svz^) in the developing human brain, which is adjacent to the ventricular zone 4. Notably, cross-species analyses have revealed anatomical and molecular discrepancies between human and mouse RL populations 5, underscoring the limitations of current mouse models of MB. The absence of effective tumor simulation models has hindered the exploration of MB tumorigenesis mechanisms and drug development. Recently, three-dimensional (3D) organoid technology has offered a promising approach for lineage tracing and therapy development in various tumors. Organoids, which are self-organized 3D tissues derived from adult or pluripotent stem cells 6, demonstrate superior preservation of native tissue architecture, functional diversity, and genomic heterogeneity compared to conventional 2D culture systems 7. Recently, glioblastoma and medulloblastoma organoids have been established directly from tumor specimens without single-cell dissociation, retaining key features of their corresponding parental tumors 8, 9. However, whether MBOs can be utilized for mechanistic studies and therapeutic evaluation remains to be further validated.

The current standard of care for MB includes surgery, radiation, and chemotherapy, with treatment strategies based on histopathological classification and molecular profiling. Although five-year survival rates range from 50% to 90%, many survivors suffer from severe neurocognitive, neurological, and psychosocial deficits due to the toxicity of standard therapies. This underscores the necessity to identify targeted therapies with reduced toxicity and improved efficacy to minimize treatment-related long-term side effects. Immunotherapy has shown significant benefits in various cancer types, but its application in the central nervous system remains challenging due to the scarcity of immunogenic antigens. TILs, a heterogeneous population of T cells within tumors, can recognize cancer-associated antigens. TILs derived from resected tumors can be expanded *ex vivo* using IL-2^10^, and reinfused into patients. Compared with other adoptive cellular therapies, such as chimeric antigen receptor T cells (CAR-T cells), TILs present unique advantages through their diverse T-cell receptor repertoire targeting multiple tumor antigens, potentially overcoming MB heterogeneity 11. Although demonstrating impressive clinical benefits in metastatic melanoma and cervical cancer, with preliminary efficacy in colorectal cancer, non-small cell lung cancer, and breast cancer 12, the clinical translation of TIL therapy for MB remains significantly challenged due to lack of preclinical models capable of recapitulating tumor heterogeneity.

In this study, we established patient-derived MBOs that faithfully preserved intratumoral heterogeneity and retained histopathological/molecular characteristics of parental tumors. MBOs exhibited remarkable invasive capabilities not only when co-cultured with human embryonic stem cell (hESC)-derived brain organoids *in vitro* but also following orthotopic or subcutaneous transplantation *in vivo*. Single-cell RNA sequencing (scRNA-seq) revealed the intertumoral heterogeneity of MBOs and uncovered the oncogenic role of MIF-CD74/CD44 pathways underlying Group 4 MB pathogenesis. Importantly, we have established a platform using MBOs to evaluate TIL cytotoxicity through integrated *in vitro* and *in vivo* approaches. Together, our findings establish patient-derived MBOs as a transformative model that bridges basic tumor biology with translational applications, promoting the development of personalized immunotherapy in medulloblastoma.

## 2. Materials and methods

### 2.1 Establishment of patient-derived medulloblastoma organoids

The clinical samples of medulloblastoma were obtained from surgical specimens provided by the Neurosurgery Department of Fudan University Affiliated Children's Hospital with informed consent from the patients. This study was approved by the Ethics Committee of Fudan University Affiliated Children's Hospital (Approval No. 2023-104) and conducted in accordance with the Helsinki Declaration.

Tumor tissue was collected from the operating room, suspended in ice cold Hibernate A (Thermo Fisher Scientific, A1247501), and transported to the laboratory on ice. The tissue was transferred to a sterile glass dish containing Hibernate A supplemented with GlutaMax (Gibco, 35050061) and penicillin/streptomycin (Gibco, 15140122) for dissection under a stereomicroscope within a laminar flow biosafety cabinet. Resected tumors were finely minced into approximately 0.5 to 1 mm diameter pieces using delicate dissection scissors, ensuring removal of necrotic or surrounding brain tissues. The tumor pieces were incubated in 1× RBC lysis buffer (Gibco, 00-4333-57) under gentle rotation for 10 minutes, then washed with Hibernate A. For histological studies, tumor pieces were fixed with 4% methanol-free formaldehyde.

The remaining tumor fragments were distributed in ultra-low attachment 6-well plates (Corning) containing 4 mL of MBO medium, which consisted of DMEM:F12 (Thermo Fisher Scientific, 10565018), Neurobasal (Gibco, 21103049), 1×GlutaMax (Gibco, 35050061), 1×Non-essential amino acid (NEAA, Gibco, 11140050), 1×PenStrep (Gibco, 15140122), 1×N2 supplement (Gibco, 17502048), 1×B27 (Gibco, 17504044) without vitamin A supplement, 1×2-mercaptoethanol (Gibco, 21985-023), and 2.5 μg/ml human insulin (Sigma, MSST0064). The plates were placed on an orbital shaker rotating at 120 rpm within a 37°C, 5% CO_2_, and 90% humidity sterile incubator. Approximately three-quarters of the medium was replenished every two days. Following a 1-2 weeks culture, dissected tumor fragments generally developed into spherical organoids. The criteria for successful establishment of patient-derived MBOs include: survival and maintenance of micro-dissected tumor fragments beyond two weeks; progressive acquisition and maintenance of characteristic spherical morphology; sustained proliferative capacity in long-term culture. For MBOs cultured for extended periods (more than one month), they were routinely cut into segments with a diameter of approximately 200-500 μm using fine dissection scissors 8.

For cryopreservation of MBOs, we developed two different protocols. First, MBOs were diced into small pieces and enzymatically digested into single-cell suspensions, with 1 mg/ml collagenase (Nordmark, S1745403). After centrifugation, the cell pellet was resuspended in MBO freezing medium supplemented with 10 μM Y-27632 (APEXBIO, B1293-1EA) and 10% DMSO (Sigma, 20-139). After being slowly cooled to -80°C with a CoolCell freezing container (ThermoFisher Scientific), frozen MBOs were transferred to liquid nitrogen for long-term storage. For recovery, frozen MBOs were quickly thawed in a 37°C water bath, centrifuged, and resuspended in MBO medium containing 10 μM Y-27632. Approximately 1×10⁴ cells were added to a well of a 96-well U-bottom low-adhesion plate, and MBO spheres were reformed within 1-2 weeks.

Alternatively, MBOs were cut into segments with a diameter of about 100 μm and incubated with MBO medium supplemented with 10 μM Y-27632 for 1 hour. Approximately 20 MBOs were resuspended in MBO freezing medium (MBO medium supplemented with 10 μM Y-27632 and 10% DMSO) in cryovials. After being kept at room temperature for 15 minutes, MBOs were slowly cooled to -80°C using a CoolCell freezing container. For recovery, vials were quickly thawed in a 37°C water bath, the freezing medium was aspirated, and MBOs were cultured in MBO medium supplemented with 10 μM Y-27632.

### 2.2 Histological and immunofluorescence staining

The fixed MBOs were resuspended in 100 μL pre-warmed 3% agarose. Tumor tissues and agarose-embedded MBOs were dehydrated and embedded in paraffin blocks, then sectioned into 5-μm-thick cross sections. For H&E staining, the slides were stained with hematoxylin and eosin for 15 min and 1 min respectively, dehydrated through graded ethanol and cleared in xylene. For immunofluorescence staining, the slides were subjected to antigen retrieval in sodium citrate buffer and permeabilized with 0.5% Triton X-100. After blocking with 10% normal goat/horse serum for one hour at room temperature (RT), tissue sections were incubated with primary antibodies overnight at 4°C and then incubated with secondary antibodies and DAPI for 1 h at RT. Specimens were imaged using an Olympus FV3000 microscope. The primary antibodies include KI67 (Gene Tech, GT2094), GFAP (Gene Tech, GT2124), SOX2 (Gene Tech, GT2163), P53 (Gene Tech, GM7001), β-Catenin (Gene Tech, GT2119), OTX2 (Proteintech, 13497-1-AP), GAB1 (Proteintech, 26200-1-AP), NeuN (Gene Tech, GT2194), and CD3 (Gene Tech, GT2002). Secondary antibodies include Alexa Fluor 647-conjugated donkey polyclonal anti-rabbit and anti-mouse IgG for respective target detection (ThermoFisher Scientific). Quantification was performed using ImageJ as follows: nuclear markers (Ki67, SOX2, OTX2, p53) were quantified by the percentage of positive nuclei; cytoplasmic markers (GFAP, GAB1) were measured by mean fluorescence intensity. β-catenin was analyzed based on its dual localization.

### 2.3 Co-culture of MBOs with hESCs derived brain organoids

The hESCs were dissociated into single cells using trypsin (Gibco, C25300054). Subsequently, 1×10⁴ cells/well were seeded in ultra-low attachment 96-well plates, and cultured in hESC medium supplemented with rock inhibitor (50 μM, InvivoChem, V2558) and FGF2 (4 ng/ml, SinoBiological, GMP-10014-HNAE) to facilitate embryoid body formation. On day 6, the embryoid bodies were transferred to ultra-low attachment 6-well plates containing neural induction medium consisting of a 1:1 mixture of DMEM/F-12 (Gibco) and Neurobasal (Gibco) medium supplemented with 0.5× N2 supplement, 0.5× B-27 supplement,5 μg/mL insulin, 2 mM L-glutamine, 0.5×NEAA, 100 μM 2-mercaptoethanol, 50 U/mL penicillin and 50 mg/mL streptomycin. On day 11, the embryoid bodies were embedded into Matrigel (Corning, 356231) and cultured in 10 cm dishes with cerebral organoid medium (B-27 supplement medium without vitamin A) for neuroepithelium formation. On day 15, the organoids were transferred to a shaker at 85 rpm. The cerebral organoid medium consisted of a mixture of N2-containing and B27-containing media (N2-containing medium: DMEM/F12, GlutaMax, 1×N2, 5 μg/mL insulin, 1 mM L-glutamine, 1×non-essential amino acids, 100 μM 2-mercaptoethanol; B27 medium: Neurobasal, 1×B27, 200 mM L-glutamine), supplemented with 50 U/mL penicillin and 50 mg/mL streptomycin. The medium was refreshed every 2 days 13. Brain organoids were labeled with red live fluorescent staining (YEASEN, 40717ES50), while medulloblastoma organoids were labeled with blue live fluorescence staining (YEASEN, 40761ES50). Subsequently, both types of organoids were co-cultured in a 96-well U-shaped low-adhesion plate with medulloblastoma organoid culture medium. Fluorescence microscopy was performed, and the extent of invasion was quantified with Image J by calculating the percentage of the cerebral organoid area infiltrated by MBOs over time.

### 2.4 Bulk RNA sequencing and transcriptomic analysis

Total RNA was extracted from both tumor tissues and organoids using the MJzol Animal RNA Isolation Kit (Majorivd) and further purified with the RNAClean XP Kit (Beckman Coulter), including a DNase treatment step (QIAGEN). RNA-seq libraries were constructed with the Illumina TruSeq Stranded mRNA kit. Specifically, mRNA was enriched from total RNA using Oligo(dT) magnetic beads, fragmented, and reverse-transcribed into cDNA with random hexamer primers. Following second-strand synthesis, the cDNA was subjected to end repair, A-tailing, adapter ligation, and PCR amplification. The quality of the libraries was evaluated on an Agilent 2100 Bioanalyzer, and sequencing was carried out on an Illumina NovaSeq 6000 platform under 150 bp paired-end mode. Raw sequencing reads were processed with Fastp to trim adapters and remove low-quality bases. Clean reads were then aligned to the human reference genome (GRCh38) using STAR (v2.6.1d) with default parameters. Gene-level read counts were generated using Rsubread and normalized to transcripts per million (TPM). Expression similarity between tissue-organoid pairs was evaluated by calculating Pearson correlation coefficients. Unsupervised hierarchical clustering was performed using established medulloblastoma (MB) subtype-specific gene sets (WNT: DKK2, TNC, EMX2, GAD1, DKK1, WIF1, SFRP1; SHH: GLI1, PTCH1, EYA1, HHIP, SFRP1, ATOH1, GLI2; Group 3: NRL, IMPG2, NPR3, MYC, OTX2, GFI1, GFI1B; Group 4: EOMES, KCNA1, KHDRBS2, RBM24, UNC5D, CDK6, SNCAIP) 14, 15, and co-expression patterns were visualized using Venn diagrams.

### 2.5 Whole exome sequencing and variant analysis

Genomic DNA was extracted from tumor tissues and organoids using the DNeasy Blood & Tissue Kit (Qiagen). Whole exome sequencing (WES) libraries were prepared with the SureSelect Human All Exon V6 kit (Agilent). Genomic DNA was sheared to 150-200 bp fragments using a Covaris S2 system, followed by end repair, A-tailing, and adapter ligation. Enriched libraries were sequenced on the Illumina NovaSeq 6000 platform with 150 bp paired-end reads. Raw sequencing data were processed with Fastp for quality control. Clean reads were aligned to GRCh38 using BWA-MEM. PCR duplicates were marked using Sambamba, and base quality recalibration was performed with GATK. Somatic mutations (SNPs and InDels) were identified using multiple callers, including Mutect2, VarScan2, and Strelka2. Variants detected by at least two callers were retained for downstream analysis. Mutation spectra and allele frequencies were compared between tissues and organoids. Unsupervised clustering was performed with the Ward method using MB-related signature genes and visualized via pheatmap (v1.0.12).

### 2.6 DNA methylation sequencing and analysis

Genomic DNA was subjected to bisulfite conversion using the Zymo EZ DNA Methylation-Gold Kit. To monitor conversion efficiency, unmethylated lambda phage genomic DNA (lambda gDNA) was spiked into each sample prior to conversion. Conversion efficiency was assessed based on the conversion rate of non-CpG cytosines within the lambda gDNA sequence, and only samples with an efficiency exceeding 99% were used for library construction and sequencing. The converted DNA was amplified, fragmented, and used to construct sequencing libraries, which were then sequenced on an Illumina platform. Raw bisulfite sequencing reads were processed with Fastp and aligned to the bisulfite-converted reference genome (GRCh38) using Bismark (v0.22.3) with Bowtie2. Methylation levels were extracted for CpG, CHG, and CHH contexts, and only sites with a coverage ≥10× were retained for further analysis. Circos plots were generated to visualize methylation density across chromosomes. Heatmaps were used to compare methylation levels of MB-associated genes and functional genomic regions, including promoters, 5'UTRs, exons, introns, 3'UTRs, and repetitive elements. The mCG/CG ratio was calculated for each region to evaluate methylation consistency.

### 2.7 Single-cell sequencing analyses of parental tumors and corresponding MBOs

Tumor tissues and MBOs were enzymatically dissociated using a papain-based neural tissue dissociation kit (Miltenyi Biotech). Single-cell suspensions were processed through the 10× Genomics Chromium platform for scRNA-seq. Libraries were sequenced as 150-bp paired-end reads on an Illumina HiSeq 4000 system. Sample demultiplexing, barcode processing, and UMI counting were accomplished through the official 10× Genomics pipeline, Cell Ranger v4.0.0. The cellranger count command was utilized to generate a gene expression matrix for each library. Reads were aligned to the hg38 human genome using STAR with default settings 16. The raw gene expression matrices were imported into R using Seurat v3.2.2. Principal component analysis (PCA) was applied to identify significant components from the dataset, and the first 30 components were further processed by means of the “TSNE” or “UMAP” function in Seurat. Graph-based unsupervised clustering was executed using the “FindClusters” function to cluster the cells 17. Malignant cells were identified from scRNA-seq data using CopyKAT^18^. Cells with a KS score > 0.1 along with large-scale chromosomal alterations were classified as aneuploid (malignant), while those with a KS score ≤ 0.1 and no evident CNVs were considered diploid (non-malignant). To minimize false positives, only cells showing consistent large-scale CNVs across at least two chromosomal arms were finally designated as malignant. The differential expression of marker genes across different clusters was determined using Wilcoxon rank-sum test.

### 2.8 Differentially expressed genes analysis and construction of a nomogram for predicting MB survival prognosis

Kyoto Encyclopedia of Genes and Genomes (KEGG) pathway enrichment analyses of differentially expressed genes were performed using the online DAVID website (https://david.ncifcrf.gov/home.jsp), with statistical significance set at *p*<0.05. Survival curves were calculated using the Kaplan-Meier method. Survival analysis was performed using clinical and transcriptomic data from the medulloblastoma cohort reported by Cavalli *et al*. (GEO accession: GSE85217), which includes 763 primary tumor samples 19. The receiver operating characteristic (ROC) curve was generated to evaluate the model. In addition, prognostic genes were screened through multivariable Cox regression; *p*<0.05 was regarded as statistically significant. The nomogram model was constructed by the rms package to predict the survival rate of patients 20. Total risk scores summarized the contributions of individual factors. All statistical analyses were performed with R software (version 4.3.2). A two-sided *p*<0.05 was considered statistically significant.

### 2.9 Co-culture of MBOs and TILs

For generation of TILs, tumors were dissected into approximately 1-3 mm³ fragments and cultured in RPMI medium supplemented with 2.05 mM L-glutamine (Gibco, 25030081), 10% FBS (Gibco, 10099141C), 55 μM 2-mercaptoethanol, 1×Penicillin-Streptomycin-Glutamine, 10 mM HEPES (Gibco, 15630080), and human interleukin-2 (IL-2, 6000 IU/mL, SinoBiological, 11848-H08H) 21, 22. TILs were grown for 2 weeks and prepared for subsequent analysis 23. FACS was performed to evaluate the populations of immune cells in expanded TILs with antibodies as follows: PE anti-human CD3 (Dakewe, 301805), FITC anti-human CD4 (Dakewe, 317407), and APC anti-human CD8 (Dakewe, 300911). Data were analyzed by FlowJo software. Approximately 5×10⁴ immune cells were seeded in a well of a 96-well plate and co-cultured with one MBO per well. Roughly half of the medium was exchanged daily by tilting the plates at a 45° angle and aspirating the medium. Apoptosis live staining dye (Yeasen, Y00001982) was added to the medium, and images were captured under a fluorescence microscope. The supernatant was collected, centrifuged at 800× g for 10 minutes to remove cells, and stored at -80°C for further analysis. The levels of TNF-α and IFN-γ in the supernatant were measured by enzyme-linked immunosorbent assay (ELISA) with a commercial kit (BD) following the manufacturer's instructions. Duplicate tests were performed for samples. Absorbance at 450 nm was measured using a microplate reader.

### 2.10 Animals and Orthotopic/Subcutaneous Transplantation of MBOs

NSG mice (4-6 weeks) were purchased from Shanghai Model Organisms Center, and housed in an SPF-grade animal facility. All animal research procedures adhered to guidelines set by Fudan University Animal Experiment Committee and approved by their protocol (2024-EKYY-034). The mice were anesthetized with 5% isoflurane (abcam, ab145581). A craniotomy of approximately 1 mm^2^ was performed above the right cerebral cortex at the intersection between the sagittal and lambdoid sutures using a micromotor drill. After removing the meninges, a single MBO was transferred into the cavity and sealed with surgical adhesive, followed by the application of dental cement (A-M SYSTEMS) onto the surrounding skull area. Animals were weighed twice a week and euthanized immediately upon weight loss or onset of neurological symptoms. Brains were carefully removed from the skull and fixed in 4% formaldehyde at 4°C overnight 8. For subcutaneous transplantation of MBOs, the skin was sterilized with 70% ethanol, a 4-mm incision was made, and a subcutaneous pocket was created using scissors. A tumor organoid was placed in the pocket, irrigated with penicillin/streptomycin solution, and the incision was closed with tissue adhesive. Animals were weighed twice weekly and euthanized after tumor formation. Subcutaneous tumor tissue was removed and fixed in 4% formaldehyde at 4°C overnight.

### 2.11 Statistical analysis

Statistical analyses were performed using GraphPad Prism version 8. A sample size of at least 3 per group was required for the test. *p*<0.05 was considered statistically significant.

## 3. Results

### 3.1 Construction of medulloblastoma organoids derived from patient tumors

To better recapitulate the histological features and cellular diversity of medulloblastoma, we established patient-derived medulloblastoma organoids (MBOs) from surgical specimens of 10 patients (Table. S1). Based on the 2016 WHO Classification of Tumors of the Central Nervous System, these MBs were classified into two WNT subtypes (MB1, MB3), four SHH subtypes (MB2, MB6, MB8, MB9), and four Group 4 subtypes (MB4, MB5, MB7, MB10). Group 3 subtypes were not included due to limitations in clinical sample availability. Subtype classification was jointly determined by histopathological assessment of tumor morphology and molecular profiling via DNA methylation analysis.

**Figure 1:**
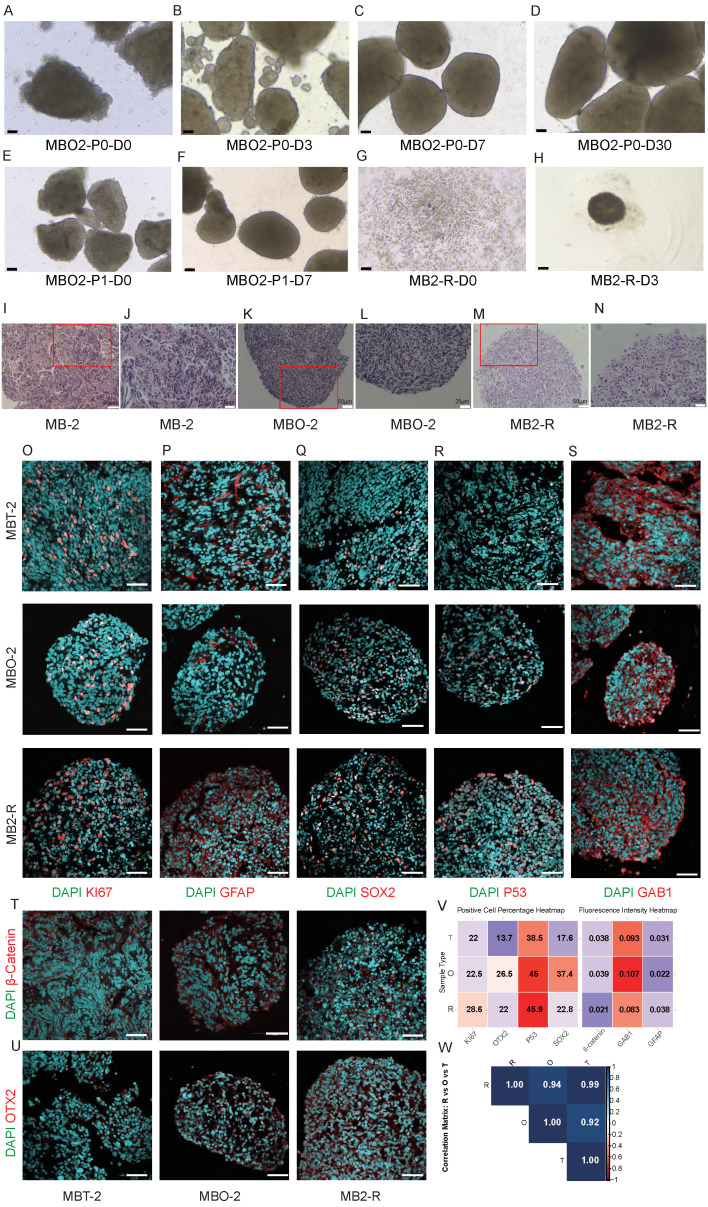
** Medulloblastoma organoids recapitulate characteristics of parental tumors.** A-D. Brightfield images of the MBOs establishment process. The MB fragments cultured on an orbital shaker gradually form organoids. Scale bars: 100 μm. E-F. Brightfield images demonstrating organoids gradually reformed after propagation by cutting them into 0.5 mm diameter pieces. Scale bars: 100 μm. G. Brightfield image of MB2 resuscitated organoids (MB2-R). Cryopreserved single cells, obtained from digested organoids, were reaggregated to form new organoids. Scale bar: 100 μm. H. Brightfield image of single cells aggregating into organoids after recovery for 3 days. Scale bar: 100 μm. I-N. Sample H&E staining images of parental tumors MB-2, corresponding MBO2 and resuscitated MB2-R. Scale bars: I and K, 50 μm; J and L, 25 μm. O-U. Representative immunofluorescence confocal images of markers (KI67, GFAP, SOX2, P53, β-Catenin, GAB1, and OTX2) in the parent tumor MB2, organoid MBO2, and MB2-R, demonstrating cellular recapitulation. Scale bar: 50 μm. V. Heatmap of marker expression in Tissue (T), Organoids (O), and resuscitated organoids (R), showing positive cell percentage (KI67, OTX2, P53, SOX2) and mean fluorescence intensity within the entire region (β-Catenin, GAB1, GFAP). W. Pearson correlation matrix between groups T, O, and R, with values closer to 1 indicating a stronger positive correlation.

Tumor fragments of approximately 1 mm³ were cultured in MBO medium under orbital shaking to enhance oxygenation and nutrient diffusion. Within 1-2 weeks, these fragments self-organized into organoids exhibiting patient-specific morphologies (Fig. 1A-D, Fig S1). To prevent central necrosis during long-term culture (>1 month), MBOs were typically cut into 200-500 µm fragments for subculturing, and these fragments regenerate into new organoids (Fig. 1E-F). Notably, organoids were successfully generated from all 10 patient samples and could be stably passaged for multiple generations. In addition, we evaluated two cryopreservation approaches. First, MBO fragments with a diameter of 100 μm were suspended in freezing medium containing Y-27632 and DMSO. After incubation at room temperature for 1 hour, the MBOs were gradually cooled to -80°C and then transferred to liquid nitrogen for long-term storage. However, approximately 1/3 of the MBOs gradually disintegrated post-recovery, likely due to incomplete DMSO penetration. Alternatively, enzymatically dissociated single cells from MBOs were cryopreserved and later reaggregated in U-bottom 96-well plates, achieving an 80% success rate in organoid reformation (Fig. 1G-H). This demonstrates the superior performance of enzymatic digestion for long-term preservation. To systematically evaluate the fidelity of MBOs in preserving the histopathological and immunophenotypic features of the original tumors, we performed comparative analyses between organoids (passages 1-2) and their matched primary tissues. H&E staining demonstrated that MBOs retained key architectural hallmarks of the parental tumors, including characteristic nuclear morphology and tissue organization across all samples (Fig. 1I-L, Fig. S2A, S2C). Notably, we performed H&E staining on resuscitated MBO2 organoids and observed that the histological architecture and nuclear morphology was retained comparable to those of the matched primary tumor and the short-term cultured organoids (Fig. 1M-N). In a representative SHH subtype case (MB2), immunofluorescence analysis revealed consistent expression patterns between the primary tumor and its derived organoids MBO2 (cultured for two weeks) and MB2-R (MB2 resuscitated organoids) for proliferation marker Ki67, glial marker GFAP, and neural progenitor marker SOX2 (Fig. 1O-Q). Moreover, high expression of SHH marker GAB1, cytoplasmic β-catenin localization and low expression of Group 4 marker OTX2 aligned with the SHH subtype designation (Fig. 1R-U). P53 mutation is a well-established high-risk factor for SHH subgroup, and positive P53 staining was observed in both the primary tissue and corresponding MBO2. Quantitative analysis of immunofluorescence markers was performed by calculating the percentage of positive nuclei for nuclear-localized markers (Ki67, SOX2, OTX2, and P53). For primarily cytoplasmic markers (GFAP, GAB1, β-catenin), the mean fluorescence intensity across all cells in the region was quantified (Fig. 1V). Quantitative analysis revealed high Pearson correlation coefficients for all markers when comparing MBO2 and MB2-R to the parental tumor (MB2), with coefficients ranging from 0.92 to 0.99 (Fig. 1W), indicating that the MBO2 and resuscitated organoid MB2-R models faithfully preserve the essential histological and cellular properties of the original MB2 tumors.

This fidelity extended across additional samples. Consistent with the above observations, Ki67, GFAP, and SOX2 were expressed to varying degrees in both organoids and primary tissues across all models (Fig. S3A, S4A). Immunofluorescence profiling confirmed the retention of subtype specific markers in MBOs: nuclear β-catenin in WNT subtype models (MB1, MB3) (Fig. S2B, S2D); high GAB1 expression in SHH subtype models (MB6, MB8, MB9) with P53 expression in MB8/MB9 (SHH-subtype, P53 wild-type) and absent P53 in MB6 (SHH-subtype, P53 mutant) matching clinical diagnoses (Fig.S3A); and elevated OTX2 expression in Group 4 subgroups (MB4, MB5, MB7, MB10) (Fig. S4A). Correlation analysis across all samples revealed strong concordance between primary tissues and their corresponding organoids (Pearson r = 0.62-0.98) (Fig. S2, S3, S4). Collectively, these results demonstrate that patient derived MBOs faithfully preserve the essential histopathological and cellular characteristics of the original medulloblastomas, thereby offering a relevant experimental model for further investigation.

To further investigate the tumorigenic potential and biological fidelity of MBOs *in vivo*, we conducted xenotransplantation experiments in immunodeficient mice using both subcutaneous and orthotopic cerebellar injection. For transplantation, intact organoids were used without dissociation to preserve the intrinsic cellular heterogeneity and three-dimensional architecture, thereby avoiding potential selection biases. For subcutaneous transplantation, an initial inoculum of approximately 30 µL of organoids developed into substantial tumors with a final volume of about 2 cm³ over three months, demonstrating rapid and consistent tumor growth. In orthotopic models, injection of 5 µL of medulloblastoma organoids into the cerebellar parenchyma led to prominent intracranial tumor growth. Within three months post-implantation, mice exhibited macrocephaly, indicating significant tumor burden. Upon euthanasia, the intracranial tumors were harvested and measured approximately 0.4 cm³ in volume. The tumor formation success rates were 100% in subcutaneous and 75% in intracranial transplantation models.

**Figure 2:**
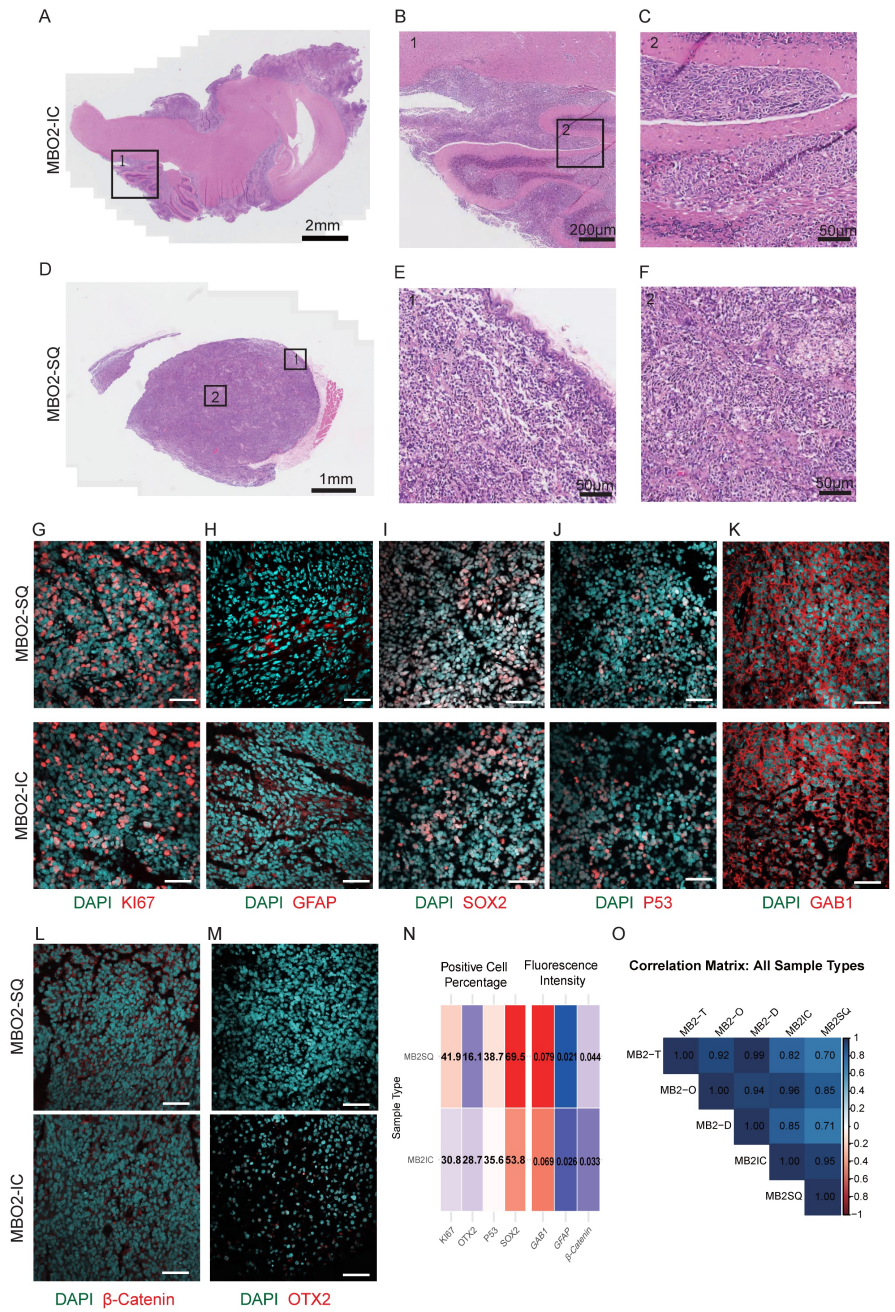
** MBOs retained the invasive capability of parental tumors.** A. Brightfield image of MBO2 intracranial (IC) xenografts with H&E staining. Scale bar: 2 mm. B. Enlarged image of box 1 in A. Scale bar: 200 μm. C. Enlarged image of box 2 in Fig. B. Scale bar: 50 μm. D. Brightfield image of MBO2 subcutaneous (SQ) xenografts with H&E staining. Scale bar: 1 mm. E. Enlarged image of box 1 in Fig. D. Scale bar: 50 μm. F. Enlarged image of box 2 in Fig. D. Scale bar: 50 μm. G-M. Confocal images of immunostaining for different markers, including KI67, GFAP, SOX2, P53, GAB1, OTX2 and β-Catenin, showing the characterization of MBO2 subcutaneous and intracranial xenografts. Scale bars: 50 µm. N. Heatmap of marker expression in subcutaneous (SQ) and intracranial (IC) xenografts. Showing positive cell percentage (KI67, OTX2, P53, SOX2) and mean fluorescence intensity within the entire region (β-Catenin, GAB1, GFAP). O. The Pearson correlation matrix depicts correlations among Tissue (T), Organoids (O), and Resuscitated Organoids (R) groups for SQ and IC. Values closer to 1 indicate a stronger positive correlation.

Histopathological examination of xenograft tumors revealed tissue organization and invasive growth patterns closely mimicking those observed in the primary medulloblastoma specimens (Fig. 2A-F). Immunohistochemical characterization confirmed the maintenance of proliferative (Ki67⁺), glial (GFAP⁺), and neural progenitor (SOX2⁺) cell populations (Fig. 2G-I), along with preserved expression of GAB1 and p53, in both transplantation models (Fig. 2J-K). Xenografts also maintained SHH-subtype hallmarks, such as cytoplasmic β-catenin and low OTX2 expression (Fig. 2L-M). Quantitative Pearson correlation analysis of immunofluorescence markers demonstrated high concordances among xenografts, organoids, and the corresponding primary tumors (r = 0.70-0.99; Fig. 2N-O). In summary, these results demonstrate that our MBO model system accurately recapitulates the histopathological features, cellular heterogeneity, and invasive growth properties of primary medulloblastoma tumors.

### 3.2 Co-culture of MBOs with hESCs derived brain organoids

**Figure 3:**
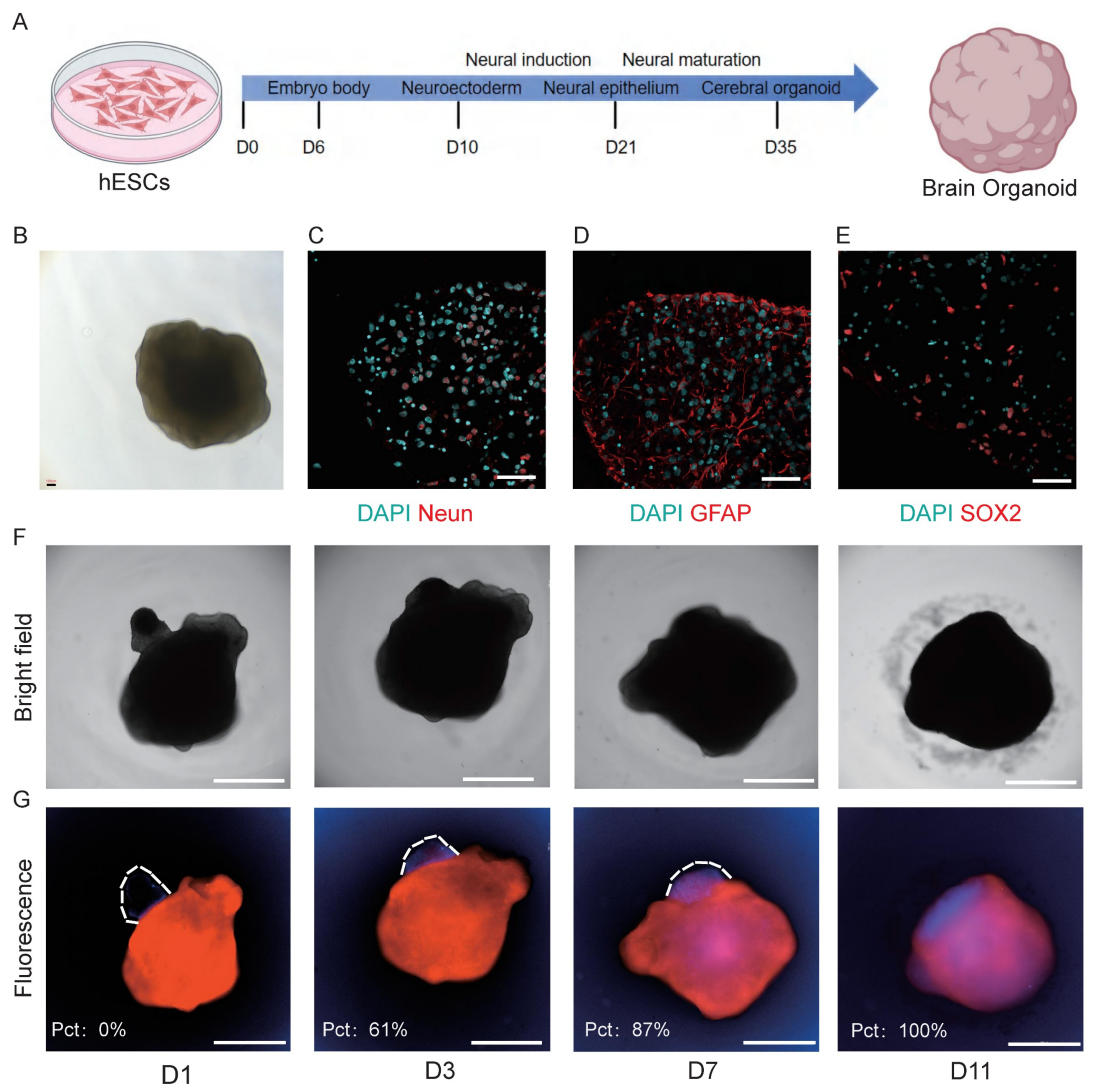
** MBO invades brain organoid.** A. Schematic diagram of the brain organoid culture process, outlining the medium and culture conditions. B. Brightfield image of brain organoid. Scale bar: 100 μm. C-E. Confocal images of immunostaining for NeuN, GFAP, and SOX2 markers, showing the characteristics of brain organoid. Scale bars: 50 μm. F. Brightfield images demonstrating that MBOs gradually penetrate brain organoids over time. Scale bars: 2000 μm. G. Fluorescence images demonstrating that MBOs (blue fluorescence) gradually penetrate brain organoids (red fluorescence) over time. The invasion ratio is quantified in the inset. Scale bars: 2000 μm.

Tumor invasion into surrounding brain tissue is a hallmark of medulloblastoma progression and a major contributor to patient mortality 24. However, studying this invasive behavior has been challenging due to the lack of physiologically relevant *in vitro* models. To address this limitation, we established a novel co-culture system combining MBOs with hESC-derived brain organoids to investigate tumor invasion. First, we generated brain organoids from hESCs and validated their neural composition by immunofluorescence staining 13, confirming the presence of neural progenitors (SOX2⁺), glial cells (GFAP⁺), and mature neurons (NeuN⁺) (Fig. 3A-E). To visualize tumor-brain interactions, brain organoids were pre-labeled with a red fluorescent dye, and MBOs were tagged with a blue live-cell tracker. The two organoid types were then co-cultured in a 96-well U-bottom low-adhesion plate to facilitate three-dimensional interactions (Fig. 3F-G). Time-course analysis revealed progressive invasive behavior. Within 24h, MBOs attached to the surface of the cerebral organoids. MBOs then gradually invaded into the brain organoids, with the invaded area reaching 61% by day 3, 87% by day 7, and culminating in complete fusion by day 11. To confirm reproducibility, we repeated the co-culture experiment in three additional MBO models (MBO4, MBO7, MBO9). All MBOs consistently invaded the cerebral organoids (Fig. S5A), achieving complete fusion varying across models at 13, 18, and 15 days, respectively (Fig. S5B), confirming the model's reproducibility. As a control, we performed additional control experiments in which cerebral organoids were labeled with distinct fluorescent tracers (red and blue) and cocultured under the same conditions as MBO-cerebral organoid assay (Fig S5C). Over a 15-day observation period, the two cerebral organoids maintained their distinct architectures and exhibited only minimal surface contact at the interface, without evidence of invasion or fusion. These experiments confirm that the fusion phenotype observed in the MBO-cerebral organoid coculture refelects the tumor-specific invasive capacity of MBOs rather than non-specific interactions between cerebral organoids. This system recapitulates the invasive behavior observed in patient tumors and provides a scalable and physiologically relevant platform for mechanistic studies. Its compatibility with 96-well plates further enables high-throughput applications such as drug screening and therapeutic target validation.

### 3.3 Multi-omics profiling demonstrates molecular fidelity in patient-derived medulloblastoma organoids

**Figure 4:**
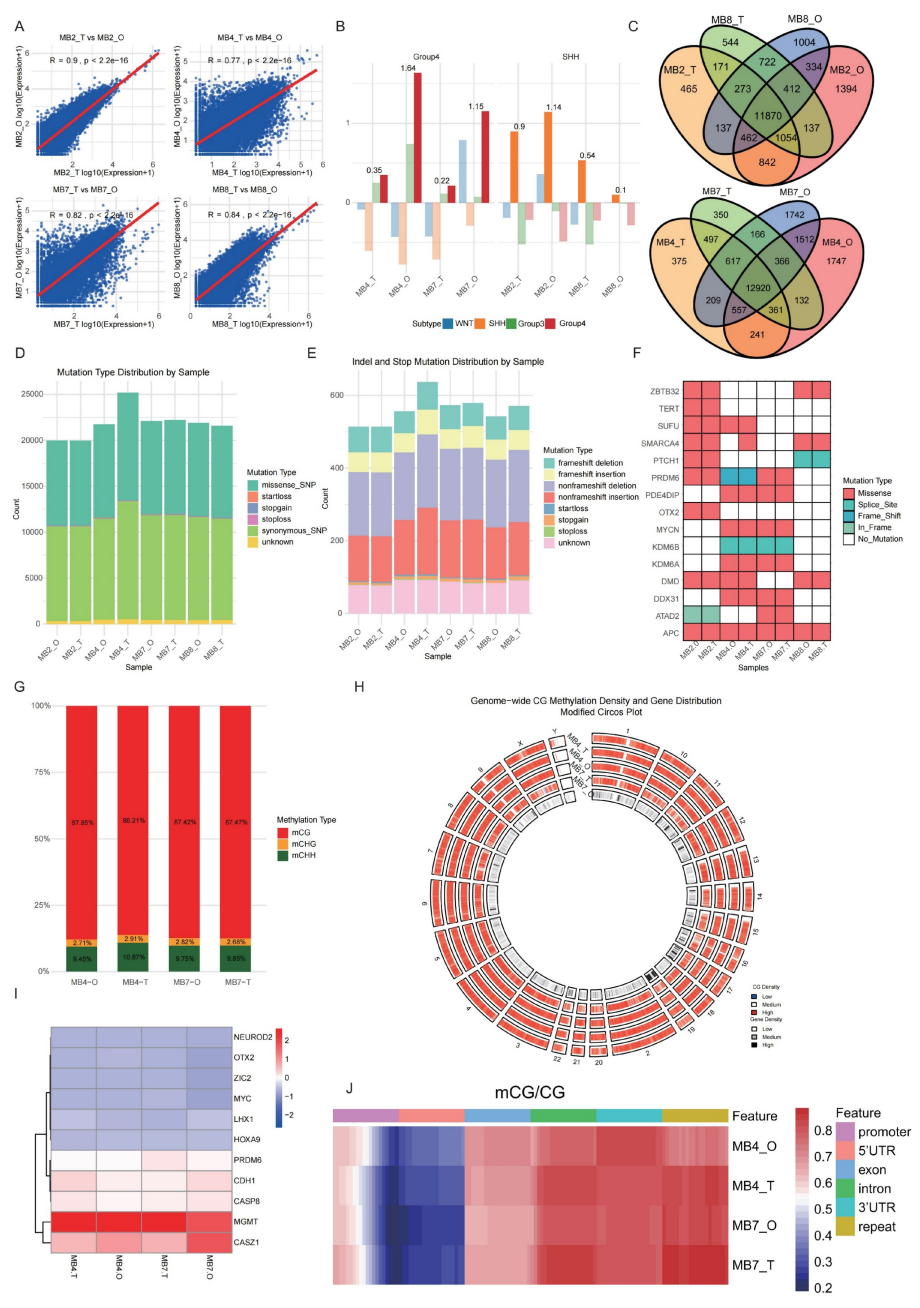
** Multi-omics characterization of molecular consistency between medulloblastoma tissues and patient-derived organoids.** A. Scatter plots showing the Pearson correlation and p-value (Pearson correlation coefficient test) of gene expression profiles between each tumor tissue (T) and its patient-derived organoid (O) for all four sample pairs (MB2, MB4, MB7, MB8). B. Unsupervised hierarchical clustering of all samples based on the expression of established medulloblastoma subtype-specific gene signatures (WNT, SHH, Group3, Group4). C. Venn diagrams illustrating the overlap of uniquely and co-expressed genes across different sample groups or subtypes. D-E. Stacked bar plot showing the distribution of insertion/deletion (InDel) and somatic single nucleotide polymorphisms (SNPs). Mutation types include synonymous, missense, stopgain, stoploss, and other mutations. Frameshift and nonframeshift InDels are the most prevalent InDel types. F. Display mutated genes associated with medulloblastoma in tissue organoid pairs. Each column represents a sample, and each row represents a gene.G. Bar plot showing the proportion of three methylation contexts (mCG, mCHG, mCHH) in MB4 and MB7 tissue-organoid pairs. H. Circos plot depicting the chromosome-wide distribution of CG methylation density. From outer to inner rings: MB4 tissue, MB4 organoid, MB7 tissue, MB7 organoid, gene density heatmap. I. Heatmap of DNA methylation levels for genes previously associated with medulloblastoma across MB4 and MB7 sample pairs. J. Heatmap comparing mCG/CG ratios across functional genomic regions (promoter, 5'UTR, exon, intron, 3'UTR, repeat) in tissue and organoid.

To verify whether medulloblastoma (MB) organoids retain the molecular characteristics of their parental tumors, we compared the consistency between paired tumor tissues and organoids cultured for two weeks using bulk RNA sequencing, whole-exome sequencing, and DNA methylation analysis. We performed transcriptome and whole-exome sequencing on samples from 4 patients (MB2, MB4, MB7, MB8) and DNA methylation sequencing on samples from 2 patients (MB4, MB7). RNA-seq results showed high expression similarity between tumor tissues and organoids, with Pearson correlation coefficients (R) ranging from 0.77 to 0.90 (Fig. 4A). Unsupervised clustering analysis based on tumor-related signature genes further revealed that tissues and organoids from the same patient clustered together, confirming subtype consistency (SHH markers enriched in MB2/MB8 pairs and Group 4 markers in MB4/MB7 pairs) (Fig. 4B). Co-expression analysis further illustrated the shared and unique gene sets among samples and subtypes (Fig. 4C).

Whole-exome sequencing revealed that organoids retained the core mutational landscape of the original tumors. We identified and annotated somatic single nucleotide polymorphisms (SNPs) and small insertions/deletions (InDels). The spectrum of mutations, including synonymous, missense, stopgain, and stoploss SNPs, as well as frameshift and non-frameshift InDels, was highly concordant between pairs (Fig. 4D-E). Notably, SHH subgroup specific mutations, such as PTCH1, SMARCA4, were preserved in tumor tissues and corresponding MBOs from MB 2 and MB 8. TERT and SUFU mutations were detected in MB2 tumors and MBOs. Group 4 representative mutations, such as KDM6A, PRDM6 were retained in tumor tissues and corresponding MBOs from MB 4 and 7 (Fig. 4F).

In DNA methylation analysis, mCG accounted for the highest proportion (86.21%-87.85%) among CG, CHG, and CHH methylation contexts (Fig. 4G). Circos plots depicting chromosome-wide methylation density revealed consistent patterns between parental tumor tissues and organoids in both MB4 and MB7 pairs (Fig. 4H). Heatmaps illustrating methylation levels of medulloblastoma (MB)-associated genes further confirmed that organoids largely preserved the epigenetic architecture of their parental tumors (Fig. 4I). For instance, in MB4 and MB7 pairs, we observed promoter hypermethylation of *CASP8*, a mechanism associated with its transcriptional silencing 25. The hypomethylation of *OTX2* is consistent with the high expression of OTX2 typical of Group 4 MBs 26. Additionally, functional region-specific methylation analysis demonstrated similar mCG/CG ratios in key genomic regions (promoters, exons, introns, UTRs, and repetitive elements) between paired tissue and organoid samples (Fig. 4J).

Together, these results demonstrate that patient-derived medulloblastoma organoids effectively retain the key genomic, transcriptomic, and epigenomic characteristics of their original tumors, including inter-tumoral heterogeneity between subtypes. This comprehensive molecular fidelity validates their utility as robust and reliable preclinical models for studying MB biology and therapeutics.

### 3.4 Unique Cell Subpopulation in Medulloblastoma

**Figure 5:**
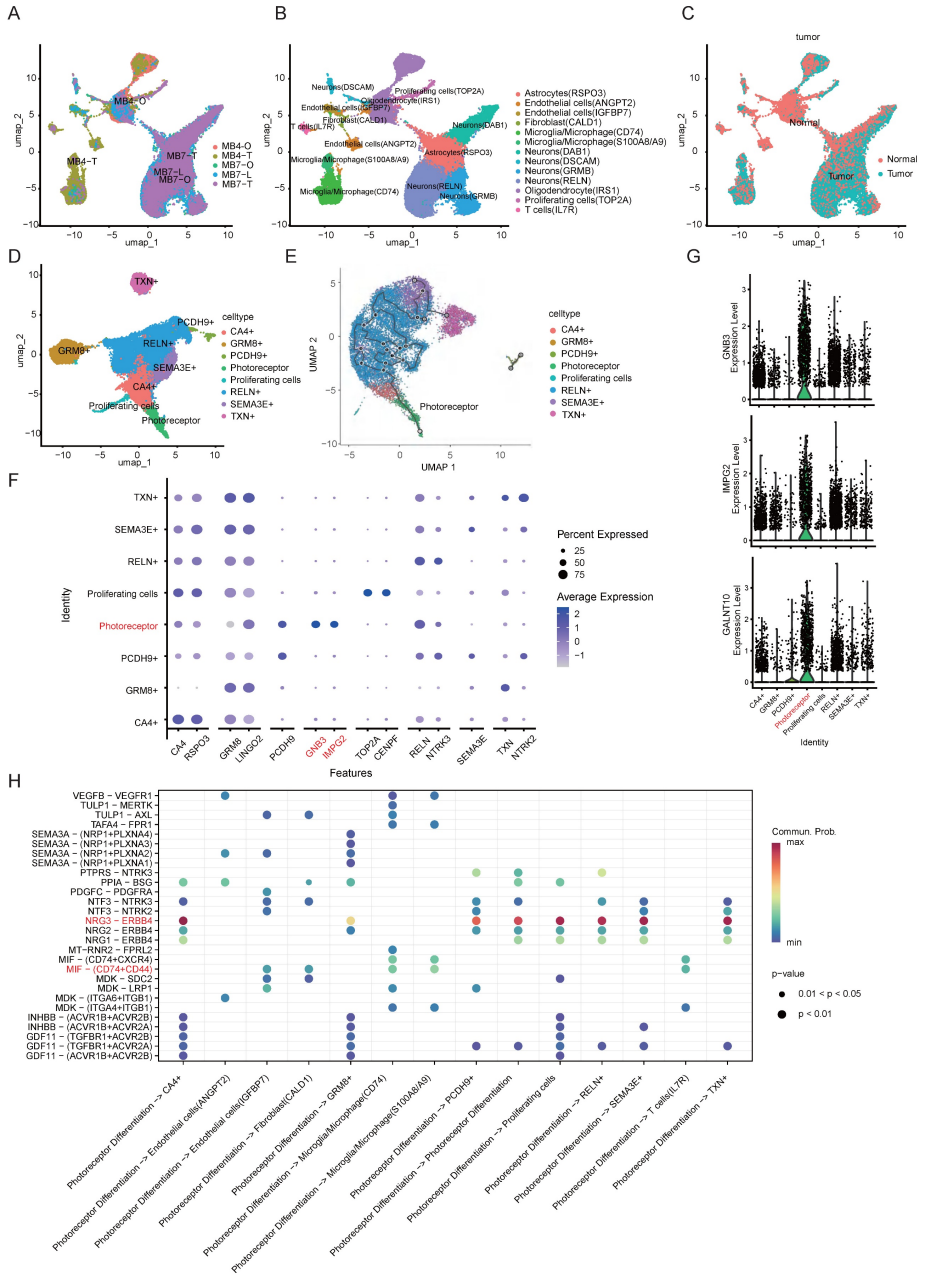
** scRNA-Seq analyses of parental tumors and corresponding MBOs.** A. t-Distributed stochastic neighbor embedding (t-SNE) of all cells derived from scRNA-seq. Cells are differentially colored according to their origin. B. t-SNE plot showing the different independent clusters obtained by integrating cells from two tumor samples and their corresponding organoids. C. Copy number karyotyping result from Copykat. D. t-SNE plot showing the different independent clusters obtained by integrating malignant cells from two tumor samples and their organoids. E. Trajectory graphs for cell groups, excluding immune and stromal subpopulations, in the “MB7-Tissue”, “MB7-O” and “MB7-L” datasets. F. Expression dot plot representing the key markers identified for each cluster of malignant cells. G. Violin plot for the expression of IMPG2, GNB3, and GALNT10 in each group of integrated malignant cells. H. Cell-cell communication network for the signaling cascades involved in interactions between the photoreceptor differentiation subgroup and other cell types.

Although Medulloblastoma Group 4 account for approximately 40% of all cases 27, they remain the least well-defined subtypes in terms of cellular origins and intratumoral heterogeneity. Bulk tumor sequencing approaches dilute the molecular profiles of rare cells such as tumor-initiating or stem-like cells. In contrast, single-cell RNA sequencing allows for complex analysis of intratumoral cellular heterogeneity. Although Group 3 and Group 4 MB were proposed to originate from the stem cell/progenitor-rich, human-specific RLVZ/RLSVZ and RLSVZ respectively, the precise developmental origin of Group 3/4 remains elusive 3, 4. To address this gap, we performed scRNA-Seq on patient-derived Group 4 medulloblastoma specimens (#4 and #7), as well as on corresponding short-term cultured MBOs (two weeks) and long-term cultured MBOs (MB7-L, three months) (Fig. 5A), with the aim of delineating cellular heterogeneity and molecular profiles. Comparative analysis revealed 8 cellular populations present across both MBOs and parental tumors, including neuronal cells, astrocytes, proliferating cells, microglia/macrophages, endothelial cells, T cells, oligodendrocyte and fibroblasts, demonstrating remarkable cellular fidelity between MBOs and original tumors (Fig. S6A, Table S3). Notably, even after extended culture, the MB7-L organoids maintained both the presence and the relative proportions of key cellular populations (Fig. 5B, Fig. S6B). To distinguish malignant and non-malignant cells, we integrated copy-number variation (CNV) inference from scRNA-seq data with biomarker expression profiling (Fig. 5C). Graph-based clustering and Uniform Manifold Approximation and Projection (UMAP) revealed eight MB subpopulations characterized by unique transcriptional signatures: CA4⁺ cells, GRM8⁺ cells, RELN⁺ cells, photoreceptor-differentiated cells, TXN⁺ cells, proliferative cells, SEMA3E⁺ cells, and PCDH9⁺ cells (Fig. 5D, Table S3).

To investigate the functional dynamics of MB subpopulations, we conducted pseudotime analysis and found that the photoreceptor differentiation subpopulation predominantly localized at the trajectory origin in Group 4 MB, revealing a distinct differentiation status compared with other subpopulations (Fig. 5E). Transcriptional profiling has revealed that MBs exhibit elevated expression of a photoreceptor differentiation program typically active in retinal tissue 28. We identified GNB3, IMPG2, and GALNT10 as specific markers for this differentiation subgroup (Fig. 5F-G). The G-protein β subunit 3 (GNB3) gene is expressed in normal retinal tissue, and its mutations are associated with retinal diseases and night blindness 29. Additionally, GNB3 polymorphisms have also been implicated in oncological prognosis, including cholangiocarcinoma, prostate and colorectal cancers 30, 31.

The IMPG2 gene encodes a protein component of the extracellular matrix critical for retinal adhesion 32, mono-allelic variants cause variably penetrant maculopathy 33. Notably, IMPG2 shows elevated expression in G4 MBs 34, and is positively regulated by the photoreceptor-specific factor CRX in Group 3 MB 35. GALNT10, a glycosyltransferase family member, promotes tumor progression by establishing an immunosuppressive microenvironment in ovarian cancer and enhances stem-like properties in platinum-resistant ovarian cancer cells 36, 37. Although the photoreceptor program is known to maintain Group 3 MBs 28, we report that an aberrant photoreceptor differentiation program, characterized by a distinct GNB3/IMPG2/GALNT10 transcriptional signature, may drive the pathogenesis of Group 4 MB. We used the core marker genes of PDS identified from scRNA-seq data (e.g., IMPG2, GNB3, CRX) for GSEA, which included GO and KEGG pathway analyses (Fig S6C-D). Notably, the GO terms include photoreceptor ribbon synapse, photoreceptor outer/inner segment, and photoreceptor cell maintenance, all of which are characteristic features of mature photoreceptors. In addition, terms such as nervous system development, glutamatergic synapse, and chemical synaptic transmission suggested the developmental origin and synaptic signaling properties of PDS. Consistently, KEGG pathway analysis showed enrichment in pathways such as glutamatergic synapse, synaptic vesicle cycle, neuroactive ligand-receptor interaction, and axon guidance. This confirms that PDS's identity as a neuronal population with photoreceptor characteristics and functional synaptic machinery.

To further investigate the functional significance of the photoreceptor differentiation subpopulation, we conducted a comprehensive cell-cell interactome analysis using CellChat. As shown in Fig. 5H, the photoreceptor differentiation subpopulation may engage in extensive intercellular communication, primary through two dominant signaling pathways: the NRG3/ERBB4 axis and the MIF-CD74/CD44 cascade. Notably, the NRG3/ERBB4 signaling pathway mediated interactions between the photoreceptor differentiation subpopulation and 7 distinct MB subpopulations. The neuroregulatory pathway, in which NRG3 activates ERBB4 through tyrosine phosphorylation, plays a pivotal role in the pathogenesis of neurological disorders and brain tumors. In Alzheimer's disease, the NRG3-ERBB4 axis mediates the interaction between astrocytes and neurons with other cell types 38. Moreover, the NRG3/ERBB4 signaling cascade has emerged as a novel therapeutic target in gliomas 39, and aberrant ERBB4-SRC signaling serves as a hallmark and oncogenic driver for group 4 MBs 40, paralleling our observed interactions. In addition, photoreceptor differentiation subpopulations may interact with microglia/macrophages, fibroblasts, and endothelial cells through the MIF-CD74/CD44 signaling cascade (Fig. 5H). MIF cytokine acts in an autocrine and paracrine manner via activating the CD74/CD44 receptors, and is linked to almost all the hallmarks of cancer 41. Moreover, MIF regulates T-cell and B-cell development, dendritic cell (DC) motility, macrophage inflammation, and thymic selection 42. This finding, coupled with our observed interaction patterns, strongly suggests the involvement of the photoreceptor differentiation subpopulation in shaping the tumor immune microenvironment. Collectively, the photoreceptor differentiation subpopulation exhibits dual roles in both oncogenesis and immunoregulation through the convergence of NRG3/ERBB4 and MIF-CD74/CD44 signaling pathways, highlighting the therapeutic potential of this subpopulation.

### 3.5 Differential gene expression analysis of PDS subpopulation relative to non-tumor and other tumor cell populations

**Figure 6:**
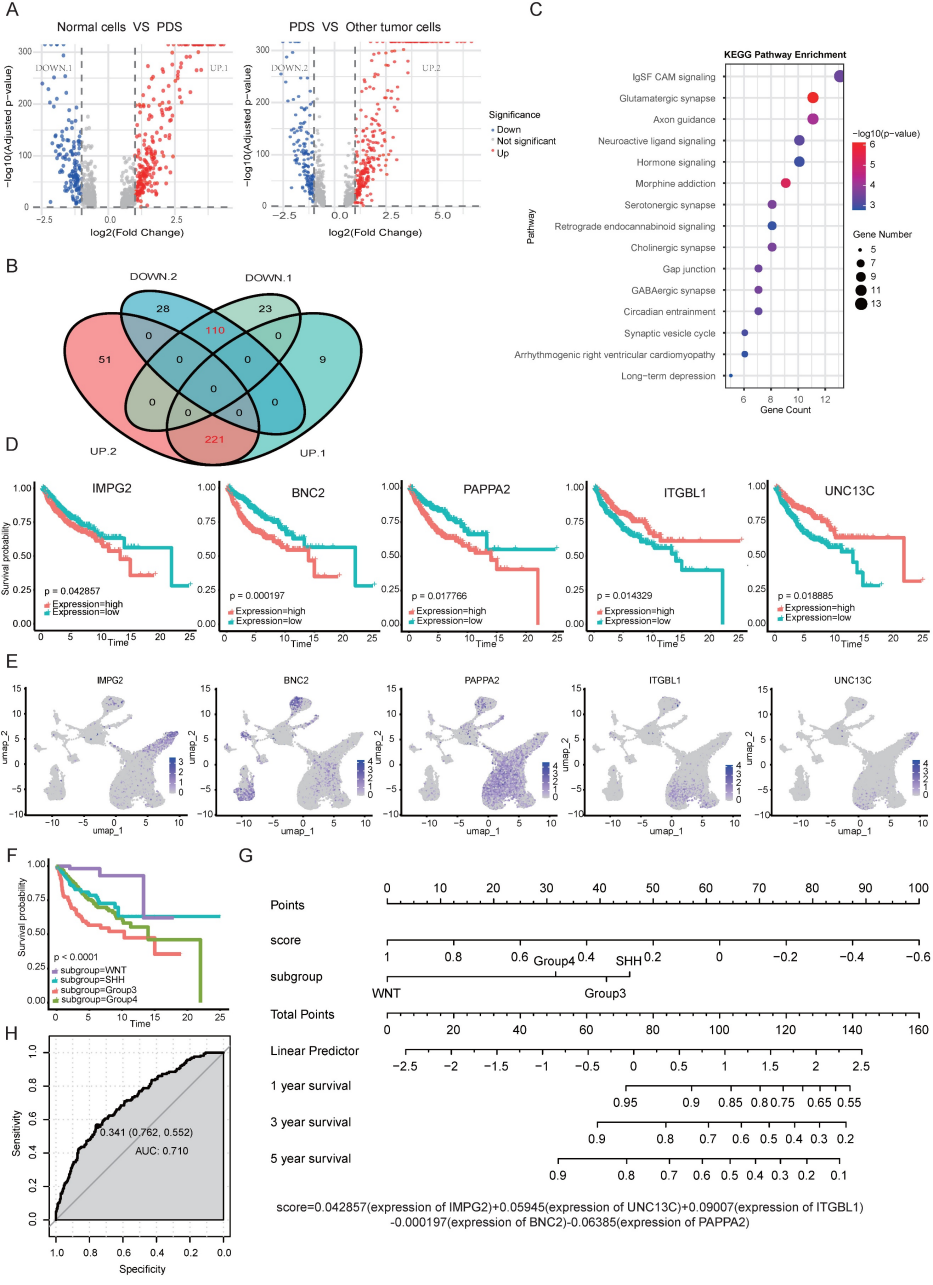
** Differential gene profiling of the photoreceptor differentiation subgroup.** A. Volcano plots of differentially expressed genes from non-tumor cells vs. PDS and PDS vs. other tumor subpopulations. B. The overlapping genes between the two gene sets (Fig. 7A) were determined using a Venn diagram. DOWN1 and UP1 indicate genes down and upregulated in normal cells vs PDS, while DOWN2 and UP2 represent genes down and upregulated in PDS vs other tumor cells. Red font indicates the overlapping regions of the two groups, referring to genes that are either upregulated or downregulated in both groups. C. KEGG analysis of upregulated differential genes, presenting the top 15 ranked pathways sorted by p-value. D-E. Survival prognosis and t-SNE plots for IMPG2, BNC2, PAPPA2, ITGBL1and UNC13C in MB patients. P values were determined by the Mantel-Cox test. F. Kaplan-Meier overall survival curves for medulloblastoma (MB) patients stratified by canonical molecular subgroups (Group 3, Group 4, SHH, WNT). P values were determined by the Mantel-Cox test. G. Nomogram predicting 1-, 3-, and 5-year OS for patients with MB. H. ROC curve evaluating the performance of the nomogram. ROC: receiver operating characteristic.

To investigate the pathogenesis of Group 4 medulloblastoma, we performed transcriptomic profiling to identify key genes associated with the photoreceptor differentiation subpopulation (PDS). First, comparing non-tumor cells to PDS revealed 230 up- and 133 down-regulated genes. Second, comparing PDS to its downstream tumor subpopulations (inferred from pseudotime analysis) identified 272 up- and 138 down-regulated genes (Fig. 6A). Taking the intersection of these two gene sets yielded 221 overlapping up-regulated genes and 110 overlapping down-regulated genes (Fig. 6B). These overlapping genes are likely critical for both the initiation and progression of Group 4 medulloblastoma. KEGG pathway enrichment analyses demonstrated that these overlapping genes were associated with key biological processes, including glutamatergic synapse, circadian entrainment, and axon guidance (Fig. 6C). The observed enrichment of glutamatergic synapse-related genes aligns with recent findings that glutamatergic synaptic input from neurons to glioma promotes tumor progression 43, suggesting potential synaptic mechanisms underlying photoreceptor differentiation-mediated MB oncogenesis.

Prognostic analysis of overlapping genes identified 36 survival-related genes, from which we constructed a multivariate regression model incorporating IMPG2, BNC2, PAPPA2, ITGBL1and UNC13C as significant predictors of MB patient outcomes (p<0.001; Fig. 6D-E). As a biomarker of the PDS subtype (Fig. 5G), IMPG2 exhibited association between high expression and poor prognosis in medulloblastoma patients (Fig. 6D). Zinc finger protein Basonuclin2 (BNC2) facilitates the transcription of Fibroblast Activation Protein Alpha (FAP) within cancer-associated fibroblasts (CAFs) and plays a critical role in angiogenesis 44. Notably, single nucleotide polymorphisms (SNPs) near BNC2 are associated with genomic dysregulation in gliobastoma 45. Integrin beta-like protein 1 (ITGBL1), a β-integrin-related extracellular matrix protein, functions as a tumor suppressor in NSCLC by modulating Wnt/PCP signaling activity 46. PAPPA2 regulates the local bioavailability of Insulin-like Growth Factor I (IGF-I), a critical driver of central nervous system (CNS) development and a process implicated in the pathogenesis of numerous cancers 47. UNC13C was characterized as a novel tumor suppressor that constrains EMT in oral squamous cell carcinoma, as demonstrated by reduced expression of EMT-associated transcription factors (Slug, Snail, Twist, ZEB1) and Vimentin, and increased Claudin1 upon its overexpression 48. The clinical relevance of UNC13C was further established in Hepatocellular carcinoma, where high expression correlated with earlier tumor stage and improved survival, validating its potential as a prognostic biomarker and therapeutic target 49.

To evaluate the prognostic significance of our five-gene signature, we conducted stratified survival analyses adjusted for key clinical variables including molecular subgroup, age, and sex. Consistent with established patterns, patients in Group 3 exhibited the worst prognosis, followed by those in Group 4 (Fig. 6F). In contrast, neither age (log rank p = 0.70) nor sex (log rank p = 0.37) was significantly associated with survival outcomes (Fig. S6E). Therefore, a multivariate Cox proportional hazards model that incorporated the five-gene signature along with clinical variables (molecular subgroup) confirmed its independent prognostic value (p<0.001). Furthermore, we constructed a nomogram to quantify the contribution of each factor and to predict 1-, 3-, and 5-year overall survival, with an AUC value of 0.710 (Fig. 6E-F). Higher total points on the nomogram were associated with worse prognosis, and could guide personalized treatment decisions for MB patients 47. Additionally, we validated the prognostic performance of the five-gene signature in an independent Group 4 cohort (n=260). The signature effectively stratified patients into distinct risk categories with significant survival differences. Notably, a higher signature was associated with improved prognosis (Fig. S6F).

### 3.6 Evaluating efficacy of TIL therapy in treating MB with MBO model

**Figure 7:**
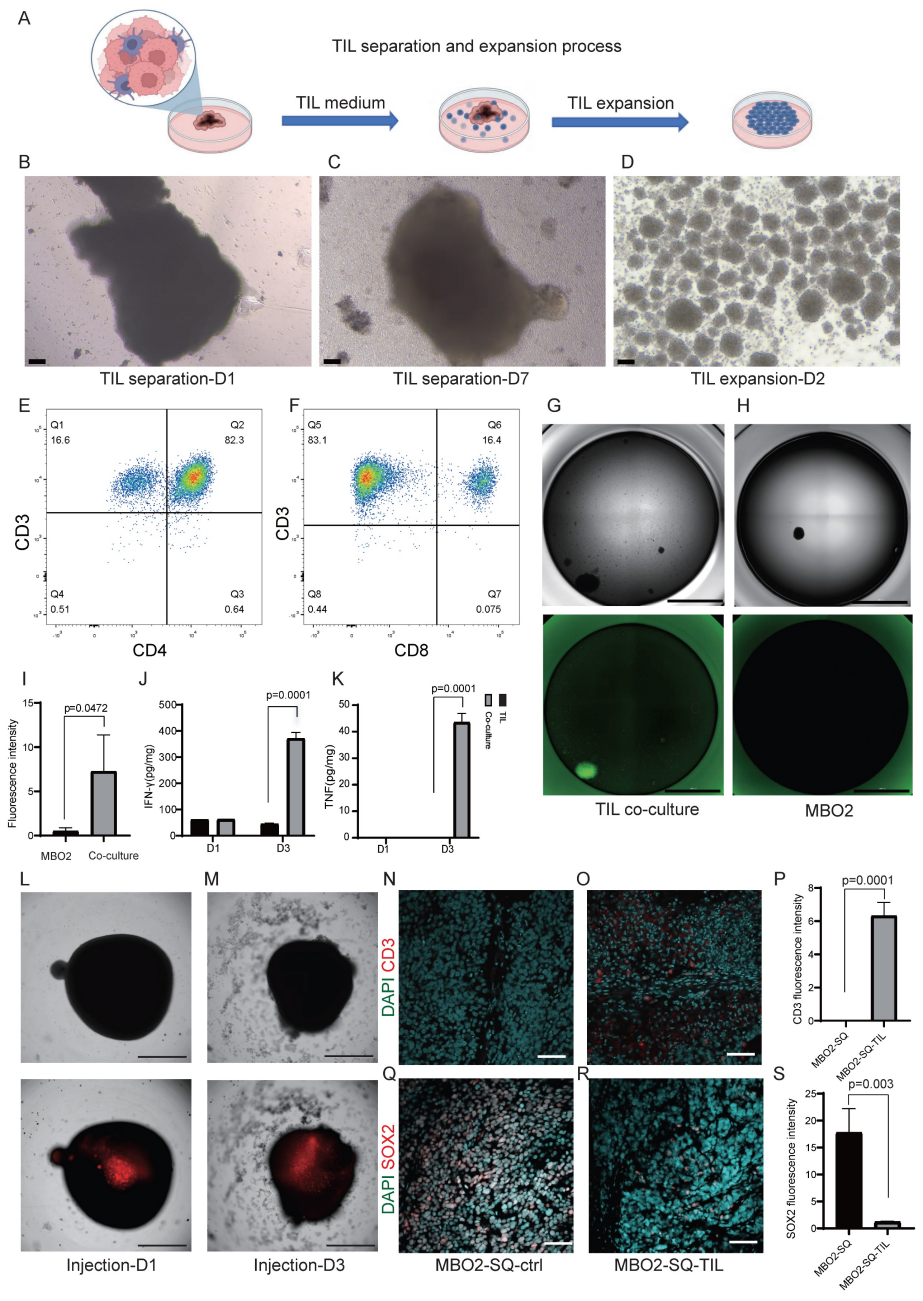
** Co-culture of TILs and MBOs.** A. Schematic diagram of the TIL isolation and expansion process. B-C. Brightfield images of the TIL isolation process. Scale bars: 100 μm. D. Brightfield image of the TIL expansion process. Scale bar: 100 μm. E-F. TILs were stained with CD3, CD4, and CD8, and then analyzed by flow cytometry. Representative FACS plots gated on CD3+ and CD4+ or CD8+ TILs are shown. G-H. Representative immunofluorescent staining of apoptotic dye in MBO2 co-cultured with TILs (G) or MBOs alone (H). Scale bars: 2000 μm. I. Statistical analysis of apoptotic fluorescence intensity in G and H, unpaired two-tailed Student's t-test. J. Statistical analysis of IFN-γ content in the supernatant, unpaired two-tailed Student's t-test. K. Statistical analysis of TNF-α content in the supernatant, unpaired two-tailed Student's t-test. L. Brightfield image and fluorescence photograph demonstrating TILs injected into the center of MBO on day 1. Scale bars: 1000 μm. M. Brightfield image demonstrating the morphological change of MBO. Fluorescence photograph indicating TILs spread around MBO 3 days after TIL injection. Scale bar: 1000 μm. N-O. Immunostaining of CD3 in MBO2 subcutaneous xenografts in mice without (N) or with TILs injection (O). Scale bars: 1000 μm. P. Statistical graph of CD3 fluorescence intensity. Q-R. Immunostaining of SOX2 in MBO2 subcutaneous xenografts in mice without (Q) or with TILs injection (R). Scale bars: 100 μm. S. Statistical graph of SOX2 fluorescence intensity in Q and R.

As an adoptive cell therapy, TILs consisting of multiple TCR clones can recognize tumor-specific neoantigens. However, the lack of appropriate models that recapitulate tumor heterogeneity has hindered the evaluation of TIL immunotherapy in treating MB. Here, we leveraged patient-derived MBOs and autologous TILs to evaluate the potential of immunotherapeutic intervention. First, TILs were expanded from surgical specimens with the addition of IL-2 for 3 weeks and gradually aggregated into spheres (Fig. 7A-D). However, prolonged culture led to a decline in proliferative capacity, consistent with aspects of T cell exhaustion. Flow cytometric analysis revealed heterogeneous immune cell composition, including both CD4+ and CD8+ T cell subsets (Fig. 7E-F). To analyze the direct killing ability of TILs, TILs were co-cultured with autologous MBOs and exhibited significant cytotoxicity, as evidenced by increased apoptotic markers (Fig. 7G-I) and elevated production of effector cytokines, such as IFN-γ and TNF-α (Fig. 7J-K). Meanwhile, we also performed replicate experiments on three organoid samples (MBO4, MBO7, and MBO9), and the results confirmed that TILs exert a cytotoxic effect on these medulloblastoma organoids (MBOs) as well (Fig.S7). To overcome limited TIL penetration in standard co-culture systems, we developed a microinjection technique for the direct delivery of 5 μL of fluorescence-labeled TILs (1×10⁵ cells) into the core of medulloblastoma organoids (MBOs) (Fig. 7L). Longitudinal tracking revealed progressive TIL distribution throughout organoids by Day 3, accompanied by characteristic apoptotic morphological changes (Fig. 7M). Together, our co-culture system offers an *in vitro* platform to evaluate the efficacy of TIL immunotherapy for treating MB.

To further validate the therapeutic responses of TILs *in vivo*, we established subcutaneous MBO-2 xenografts following administration of 1×10⁷ TILs through tail vein injection. Immunohistochemical analysis demonstrated significant TIL infiltration in MBO xenografts, as confirmed by CD3-positive staining (Fig. 7N-P). Moreover, the percentage of SOX2-positive cells was reduced in TIL-treated xenografts compared with controls (Fig. 7Q-S), suggesting that infiltrated TILs suppressed the proliferative activity of tumor cells in MBO xenografts. Collectively, our findings highlight the translational potential of TIL immunotherapy and demonstrate the utility of MBO models for personalized immunotherapy testing in medulloblastoma.

## 4. Discussion

### 4.1 MBOs as a novel model to recapitulate medulloblastoma characteristics

Despite advances in neurosurgery, chemotherapy, and radiotherapy, patients with brain tumors continue to experience limited clinical benefits. A critical challenge lies in the lack of biologically relevant models that accurately recapitulate tumor characteristics, thereby hindering mechanistic investigations, therapeutic development, and precision drug screening. This limitation is particularly evident in medulloblastoma, a highly heterogeneous malignancy recognized in the updated WHO classification as comprising distinct molecular subgroups. These subgroup-specific characteristics necessitate the development of corresponding experimental models to facilitate mechanistic research and clinical trials.

Conventional MB models, including immortalized cell lines, patient-derived xenografts (PDX), genetically modified cerebellar precursors, and mouse models, each exhibit limitations in replicating the cellular origins, genetic landscapes, and therapeutic responses of parental tumors. To address these challenges, we generated MBOs by mechanical fragmentation of patient-derived tumor tissue, preserving native tumor architecture more effectively than enzymatic dissociation methods. MBO establishment typically required less than 7 days, with morphological variations across different samples (Fig. S1), reflecting intrinsic tumor heterogeneity. We successfully generated MBOs from 10 patients representing three major molecular subtypes (WNT, SHH, Group 4), achieving 100% establishment efficiency and stable passage. These established organoids maintained proliferative capacity for more than 6 months in culture, and demonstrates remarkable fidelity across extended propagation. Moreover, we modified the biobanking strategy by enzymatically dissociating MBOs into single-cell suspensions, which reliably reformed organoids upon thawing (Fig. 1G-H). Histopathological and immunohistochemical analyses confirmed that MBOs retained the histological architecture and cellular composition of the parental tumors (Fig. 1I-U). High correlation coefficients in marker expression between tumors and organoids further validated the model's reliability (Fig. 1V-W).

Beyond histology and cellular composition, multi-omics profiling confirmed that MBOs preserve the genomic, transcriptomic, and epigenomic landscapes of the original tumors (Fig. 4). The retention of subtype-specific somatic mutations, alongside global transcriptomic and DNA methylation patterns, form a cornerstone of our validation. This comprehensive molecular fidelity ensures that findings from MBO-based studies are directly relevant to the human disease, making MBOs as physiologically relevant and superior preclinical model compared to traditional cell lines.

MBOs demonstrate infiltration capabilities in both *in vitro* and *in vivo* contexts, as shown by co-culture with hESC-derived cerebral brain organoids (Fig. 3F-G) and following orthotopic or subcutaneous transplantation (Fig. 2A-F). Xenografted MBOs accurately preserve the histological features, cellular identities, and invasive characteristics of their parental tumors (Fig. 2G-O). Notably, the co-culture model combining brain organoids with medulloblastoma organoids (BO-MBOs) achieves the highest fidelity in mimicking clinical tumor invasion dynamics *in vitro*. Future work will characterize the invasive phenotype further by assessing basement membrane integrity (e.g., via immunostaining for Laminin or Collagen) and quantifying the levels and activity of key Matrix Metalloproteinases (MMP-2 and MMP-9). This system not only enables detailed investigation of tumor-microenvironment interactions but also provides a scalable platform amenable to future high-throughput drug screening applications.

A key limitation of the present study is the lack of patient-derived organoid models from Group 3 medulloblastoma, a highly aggressive and clinically high-risk subtype. This absence is mainly due to the limited availability of surgical specimens from this subgroup. As a result, the current biobank does not fully capture the heterogeneity of medulloblastoma, which may limit its utility for studies focused on high-risk disease and subtype-specific therapeutic vulnerabilities. In future work, we will prioritize the collection of Group 3 clinical samples and aim to establish corresponding organoid models. Incorporation of Group 3 models will be essential for building a more comprehensive and representative preclinical platform for medulloblastoma research. Although MBOs preserve key molecular and histological features of patient tumors and offer advantages over traditional cell line models, they cannot fully recapitulate several critical aspects of the *in vivo* tumor microenvironment (TME), particularly the functional vascular network and the diversity of immune cell populations, such as macrophages, dendritic cells, and B cells, which are important for tumor-microenvironment interactions 50. As a result, the model has limited capacity to fully mimic the complexity of the *in vivo* TME and to capture microenvironment-dependent biological responses. To address these gaps, future studies will focus on engineering vascularized MBOs through co-culture with endothelial and perivascular cells, as well as reconstruction of the immune microenvironment by incorporating patient-derived immune cells. These efforts could help establish an organoid-microenvironment co-culture model that more closely resembles the *in vivo* state and further improves the utility of MBOs for investigating tumor biology and therapeutic responses.

### 4.2 Photoreceptor program in Group 4 medulloblastoma

MB exhibits significant intra- and intertumoral heterogeneity across subtypes and patients, manifested through malignant cellular hierarchies and diverse tumor microenvironments. Tumorigenesis and recurrence mechanisms are driven by the diversity of tumor-initiating cells and their dynamic interactions with the tumor microenvironment. Single-cell technologies have revolutionized our understanding of MB tumorigenesis, particularly revealing distinct cellular origins for different subgroups: the WNT subgroup of MBs originates from extracerebellar lower rhombic lip cells, while SHH tumors arise from cerebellar granule neuron progenitors (GNPs)^3^. Although the origins of group 3/4 MBs have been less characterized due to limited animal models, recent advances have demonstrated overlapping molecular signatures between these subgroups. Notably, over 50% of group 3 MBs maintain a photoreceptor differentiation program critical for tumor maintenance 28. Emerging evidence suggests a unified rhombic lip origin of group 3 and group 4 MBs, which display photoreceptor and unipolar brush cell expression profiles 3. In this study, our single-cell sequencing analysis revealed that MBOs accurately recapitulate parental tumor heterogeneity (Fig. 5A-D). Pseudotime trajectory analysis positioned the photoreceptor differentiation subpopulation at the developmental origin of group 4 MBOs/MBs (Fig. 5E), characterized by elevated expression of GNB3, IMPG2, and GALNT10 (Fig. 5F-G). Supporting these findings, clinical correlation analysis of published datasets confirmed that IMPG2 overexpression is negatively associated with disease progression and patient survival (Fig. 6D). DEGs analysis between PDS cells relative to non-tumor and other tumor cell populations implicated key pathways, including glutamatergic synapse regulation, circadian entrainment, and axon guidance (Fig. 6B). These findings align with established neurobiological principles, as glutamatergic signaling through Ca²⁺-permeable AMPA receptors is known to promote glioblastoma migration and proliferation. Multiple microarray analyses have linked circadian entrainment and glutamatergic pathways to pediatric MB pathogenesis 51, 52. The critical role of the photoreceptor program in group 4 MB development warrants further mechanistic investigation. To facilitate clinical trials, we developed a prognostic nomogram incorporating expression levels of IMPG2, BNC2, PAPPA2, ITGBL1and UNC13C expression levels with clinical covariates (molecular subgroups), demonstrating significant predictive value for patient outcomes (Fig. 6D-G), as supported by independent Group 4 cohort (Fig. S6F).

PDS is a stable malignant cluster with progenitor-less-differentiated-like transcriptional features. It was consistently detected in primary tumors as well as short-term (2-week) and long-term (3-month) MBOs. Notably, cell-cell communication analysis revealed that the photoreceptor differentiation subgroup actively interacts with other MB subpopulations via the NRG3/ERBB4 signaling cascade (Fig. 5I), highlighting its role as a core regulator influencing tumor behavior. Simultaneously, this subgroup modulates microglia/macrophages through the MIF-CD74/CD44 signaling cascade (Fig. 5H), suggesting that the photoreceptor differentiation subgroup may drive malignancy by interacting with the tumor microenvironment. These findings collectively underscore the pivotal role of photoreceptor programming in the pathogenesis of group 4 MB, while emphasizing the necessity of mechanistic exploration into these newly identified signaling networks and their interactions with the tumor microenvironment.

### 4.3 MBOs model for evaluating TILs immunotherapy efficacy

Current standard therapy for MB combines maximal surgical resection with radiotherapy and chemotherapy. Despite this intensive multimodal approach, approximately 30% of patients experience treatment failure and relapse, with 5-year post-relapse survival rates below 10%. Recently, immunotherapy has emerged as a promising alternative. Various immunotherapeutic strategies, such as immune checkpoint inhibition and CAR T-cell therapy, have been explored using *in vitro* and* in vivo* models, as well as clinical investigations 53. In this study, we expanded TILs from MB surgical fragments and co-cultured them with MBOs *in vitro*. TIL cytotoxicity is primarily mediated by CD8+ T cells through the production of IFN-γ and TNF-α (Fig. 7J-K). Subsequent *in vivo* validation using patient-derived organoid-based xenografts (PDOX) models demonstrated enhanced CD3+ immune infiltration and a significant reduction in SOX2+ medulloblastoma cells following TIL administration (Fig. 7N-S), confirming the *in vivo* tumor-killing effects of TILs. These findings support the potential of TILs as a promising immunotherapy for MB.

Recent research has focused on immune checkpoint targets, such as B7-H3, which is differentially expressed in pediatric MB, particularly in Group 4 tumors 53. Additionally, CD276 is overexpressed across all MB subgroups, and CD24 is significantly elevated in SHH, Group 3, and Group 4 subgroups compared with non-tumor tissues 54. Whether combining TILs with checkpoint inhibitors (e.g., PD-1, B7-H3, and CD276 blockers) enhances therapeutic efficacy remains to be systematically validated using our MBO model. Another limitation is T cell exhaustion, which arises from chronic antigen exposure and is characterized by reduced proliferation and upregulation of inhibitory receptors 55. Multi-omics studies have shown that T cell exhaustion represents a stable state with altered transcriptional and epigenetic profiles 56. Targeting epigenetic regulators during CD8+ T cell differentiation is a promising strategy to counteract exhaustion. Recent studies have demonstrated that disrupting epigenetic modulators, such as DNA methyltransferase Dnmt3a, DNA demethylation enzyme Tet2, and polycomb group repressive deubiquitinase complex Asxl1, in CD8+ T cells can prevent exhaustion and enhance therapeutic durability and responsiveness 57. In the future, our established MBO-TIL co-culture model could be used to screen and identify epigenetic modulators that resist T cell exhaustion or preserve stem cell-like T cell populations.

While our MBO-TIL co-culture system provides a valuable platform for assessing autologous T cell cytotoxicity, it is important to note its limitations in recapitulating the full complexity of the native tumor immune microenvironment (TIME). Standard MBO cultures lack endogenous immune populations such as tumor-associated macrophages, microglia, dendritic cells, and myeloid-derived suppressor cells, as well as physiologic stromal and cytokine networks. These elements are critical for modulating antigen presentation, T cell priming, exhaustion, and overall immune response dynamics *in vivo* 58. Future efforts incorporating additional immune cell populations, cytokine modulation, and other components of the tumor immune microenvironment may help improve its physiological relevance for immunotherapy studies. In addition, our TIL-MBO co-culture and xenograft experiments primarily assess the direct cytotoxic effects of TILs on tumor cells, rather than systemic immune processes such as T-cell trafficking, memory formation, or interactions with peripheral immune organs. Therefore, more complex models are still required to evaluate therapeutic durability and combination strategies involving systemic immune regulation, such as TIL therapy combined with immune checkpoint inhibitors (e.g., anti-PD-1/PD-L1) or other immunomodulatory agents. In this context, humanized mouse models with a reconstituted human immune system may provide a more suitable platform for assessing both long-term efficacy and systemic immune responses 59. Therefore, future work will combine the MBO model with humanized mouse models to bridge this gap, enabling a comprehensive assessment of both direct tumor killing and systemic immune modulation.

In summary, we have established patient-derived MBOs that faithfully recapitulate the histopathological and molecular features of their parental tumors, as demonstrated by integrated immunofluorescence staining and multi omics sequencing data. The BO-MBO system enables comprehensive investigation of tumor-microenvironment interactions and invasive mechanisms. Through single-cell sequencing, we identified a photoreceptor differentiation subpopulation that mediates intercellular communication and contributes to Group 4 MB pathogenesis. Importantly, our MBO platform serves as a robust tool for assessing TIL-based immunotherapies for MB. Subsequent work integrating myeloid co-cultures, immune checkpoint inhibitors, or cytokine supplementation will be essential to build more physiologically relevant models for immunotherapy testing.

## Supplementary Material

Supplementary figures and tables 1-2.

Supplementary table 3.

## Data Availability

All raw sequencing data reported in this paper have been deposited in the Genome Sequence Archive (GSA). Specifically, the single-cell RNA sequencing (scRNA-seq) data are available under Project ID: PRJCA029327, PRJCA058601, and the transcriptomic, whole-exome, and DNA methylation sequencing data are available under Project ID: PRJCA052783.
