# Supplementary material for: Elucidating Tumorigenesis Mechanisms and Assessing Immunotherapeutic Efficacy in Patient-Derived Medulloblastoma Organoid Models
Source: Int J Biol Sci. 2026 Apr 23;22(9):4878–99. doi: 10.7150/ijbs.116040 (PMC13182545; doi:10.7150/ijbs.116040)
Supplement: Supplementary file 1 — Supplementary figures and tables 1-2. [file ijbsv22p4878s1.pdf]

Figure S1. Brightfield images of organoids established from parental tumors.

Figure S2. Histological and β-Catenin expression profiling in medulloblastoma organoids and matched tumors.

A, C. Representative hematoxylin and eosin (H&E) staining of parental tumor tissues and their matched patient-derived organoids (MB1, MB3-MB10). Scale bars: 50 μm. B, D. Representative immunofluorescence confocal images of β-Catenin expression in the parental tumors and their matched MBOs (MB1, MB3-MB10). Scale bars: 50 μm. E. Bar graph comparing the overall intensity of β-Catenin in patient-derived organoids versus original tumor tissues across the MB sample series (MB1, MB3-MB10). Values represent mean fluorescence intensity measurements within the entire region. Scatter plot showing the correlation of β-Catenin overall intensity between patient-derived organoids and their corresponding original tumor tissues for each MB sample. The Pearson correlation coefficient (R) and p-value (Pearson correlation coefficient test) are indicated, demonstrating a statistically significant positive correlation. F. Bar graph comparing the positive cell percentage of β-Catenin in patient-derived organoids versus original tumor tissues across the MB sample series (MB1, MB3-MB10). Scatter plot showing the correlation of β-Catenin positive cell percentage between patient-derived organoids and their corresponding original tumor tissues for each MB sample. The Pearson correlation coefficient (R) and p-value (Pearson correlation coefficient test) are indicated, demonstrating a statistically significant positive correlation.

Figure S3. Expression and correlation analysis of KI67, P53, and GAB1 in patient-derived medulloblastoma organoids and matched parental tumors.

A. Representative immunofluorescence confocal images of KI67, P53, and GAB1 expression in parental tumors and their matched MBOs (MB1, MB3-MB10). Scale bars: 50 μm. B–D. Quantitative comparison of KI67, P53, and GAB1 expression between MBOs and parental tumors. For each marker, upper panels show bar graphs comparing the respective measurements，percentage of KI67-positive cells, percentage of P53 positive cells, and overall GAB1 intensity，in MBOs versus original tumor tissues across the MB series. Lower panels display scatter plots illustrating the correlation of these measurements between MBOs and matched parental tumors for each sample.

Pearson correlation coefficient (R) and p-value (Pearson correlation coefficient test) are indicated.

Figure S4. Expression and correlation analysis of OTX2, SOX2, and GFAP in patient-derived medulloblastoma organoids and matched parental tumors.

A. Representative immunofluorescence confocal images of OTX2, SOX2, and GFAP expression in parental tumors and their matched MBOs (MB1, MB3-MB10). Scale bars: 50 μ m. B–D. Quantitative comparison of OTX2, SOX2, and GFAP expression between MBOs and parental tumors. For each marker, upper panels show bar graphs comparing the percentage of OTX2-positive cells (B), percentage of SOX2-positive cells (C), and overall GFAP intensity (D) in MBOs versus original tumor tissues across the MB series. Lower panels display scatter plots illustrating the correlation of these measurements between MBOs and matched parental tumors for each sample. Pearson correlation coefficients (R) and corresponding p-values (Pearson correlation coefficient test) are indicated for each marker.

Figure S5. Tumor invasion of MBOs into brain organoids in a co-culture model.

A. Brightfield and corresponding fluorescence images showing the progressive infiltration of medulloblastoma organoids (MBOs, red) into brain organoids (blue) over time. The experiment was repeated in MBO4, MBO7, and MBO9 with consistent results. Scale bars: 2000 μm. B. Faceted line plots depict the invasion ratio (0-1.0) of four distinct MBO lines (MBO2, MBO4, MBO7, MBO9) into brain organoids across co-culture period. The X-axis represents time (in days), and the Y-axis represents the quantitative invasion ratio. C. Brightfield and corresponding fluorescence images illustrate the co-culturing results of red-labeled and blue-labeled cerebral organoids. Scale bars: 2000 μm.

Figure S6. Expression Profiles of Key Markers and associated Survival Prognosis in MB

A. Expression dot plot representing the key markers identified for all cells. B. Proportions of major cell types, annotated by key marker genes. C. GO analysis of PDS core marker genes, showing the top 20 enriched pathways. D. KEGG analysis of PDS core marker genes, displaying the top 20 ranked pathways. E. Two-panel Kaplan-Meier survival curves stratified by patient age (Old vs. Young; left) and gender (F vs. M; right).

P values were determined by the Mantel-Cox test. F. Kaplan-Meier survival curve for Group 4 medulloblastoma patients stratified by high versus low risk score. P values were determined by the Mantel-Cox test.

Figure S7. Apoptosis and Cytokine Secretion in MBO-TILs Co-Culture System

A. Representative immunofluorescence (green, apoptotic dye) and matched bright-field images of MBOs co-cultured with autologous TILs or cultured alone. Images derived from independent replicate experiments MBO4, MBO7 and MBO9. Scale bar: 2,000 μm. B. Quantification of apoptotic signal intensity, in the same replicate sets (MBO4, MBO7, MBO9). C-D. Quantification of (C) IFN-γ and (D) TNF-α concentrations in culture supernatants of the same replicate sets (MBO4, MBO7, MBO9).

Table S1. Sample information of medulloblastoma organoids.

Table S2. Immunohistochemical Comparison Between 10 MBOs and Corresponding Primary Tumors.

Table S3. scRNA-seq Cluster Annotation and Marker Genes.

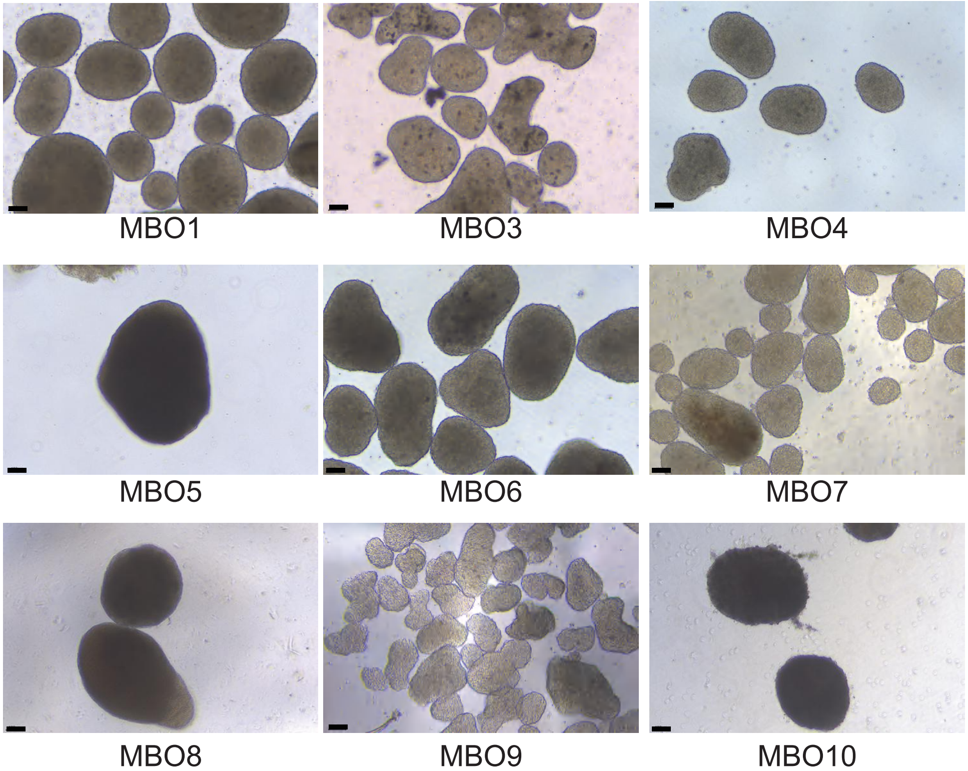
MBO1
MBO3
MBO4
MBO5
MBO6
MBO7
MBO8
MBO9
MBO10

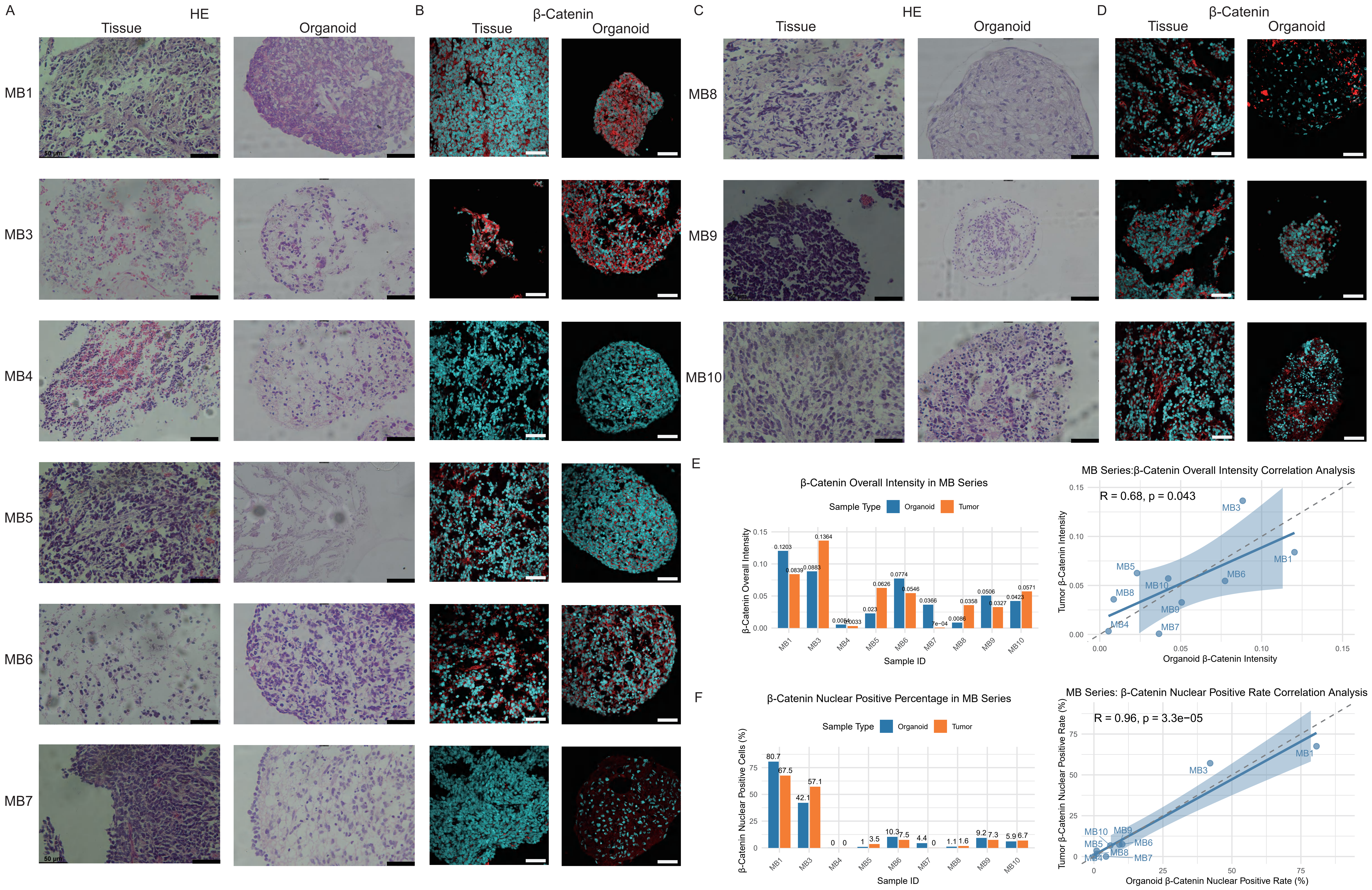

A
HE
Tissue
Organoid
B
β-Catenin
Tissue
Organoid
C
HE
Tissue
Organoid
D
β-Catenin
Tissue
Organoid
MB1
MB3
MB4
MB5
MB6
MB7
MB8
MB9
MB10
50 μm
E
β-Catenin Overall Intensity in MB Series
Sample Type
Organoid
Tumor
β-Catenin Overall Intensity
Sample ID
MB Series:β-Catenin Overall Intensity Correlation Analysis
R = 0.68, p = 0.043
Tumor β-Catenin Intensity
Organoid β-Catenin Intensity
F
β-Catenin Nuclear Positive Percentage in MB Series
Sample Type
Organoid
Tumor
β-Catenin Nuclear Positive Cells (%)
Sample ID
MB Series: β-Catenin Nuclear Positive Rate Correlation Analysis
R = 0.96, p = 3.3e−05
Tumor β-Catenin Nuclear Positive Rate (%)
Organoid β-Catenin Nuclear Positive Rate (%)

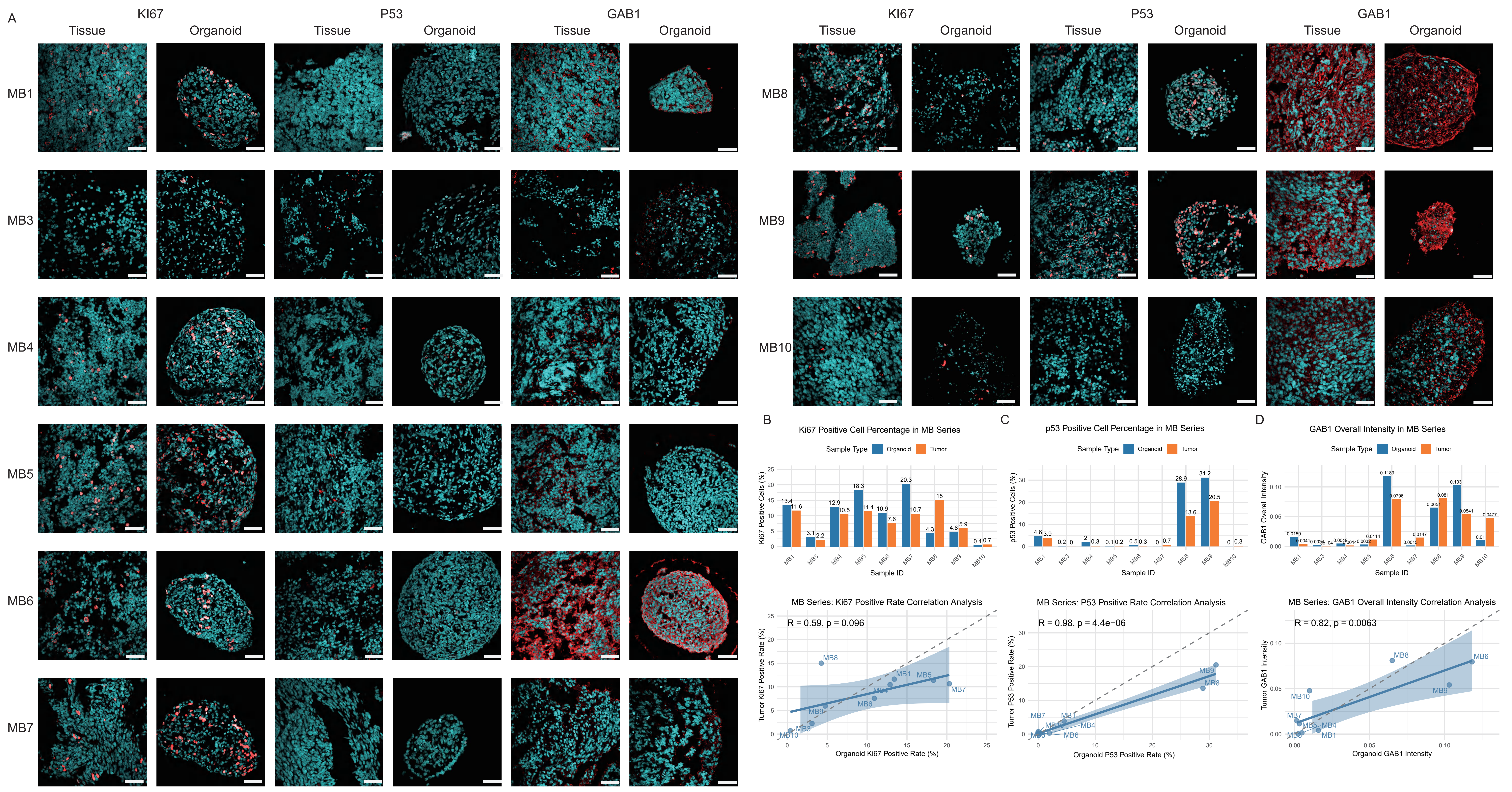
A
KI67
P53
GAB1
Tissue
Organoid
MB1
MB3
MB4
MB5
MB6
MB7
MB8
MB9
MB10
B
Ki67 Positive Cell Percentage in MB Series
Sample Type
Organoid
Tumor
Ki67 Positive Cells (%)
Sample ID
C
p53 Positive Cell Percentage in MB Series
p53 Positive Cells (%)
D
GAB1 Overall Intensity in MB Series
GAB1 Overall Intensity
MB Series: Ki67 Positive Rate Correlation Analysis
R = 0.59, p = 0.096
Tumor Ki67 Positive Rate (%)
Organoid Ki67 Positive Rate (%)
MB Series: P53 Positive Rate Correlation Analysis
R = 0.98, p = 4.4e−06
Tumor P53 Positive Rate (%)
Organoid P53 Positive Rate (%)
MB Series: GAB1 Overall Intensity Correlation Analysis
R = 0.82, p = 0.0063
Tumor GAB1 Intensity
Organoid GAB1 Intensity

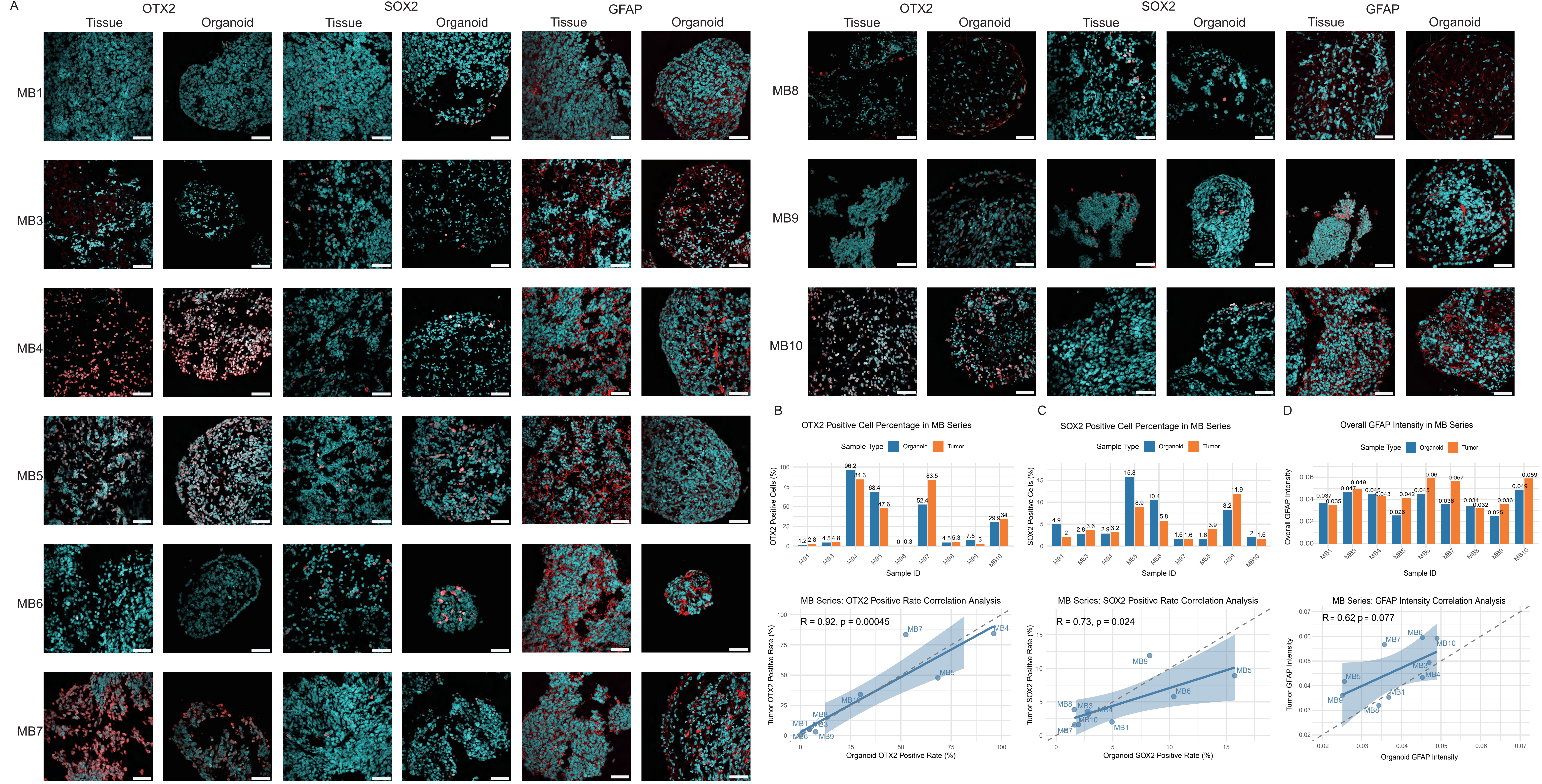

A
OTX2
SOX2
GFAP
Tissue
Organoid
MB1
MB3
MB4
MB5
MB6
MB7
MB8
MB9
MB10
B
OTX2 Positive Cell Percentage in MB Series
Sample Type
Organoid
Tumor
OTX2 Positive Cells (%)
Sample ID
C
SOX2 Positive Cell Percentage in MB Series
SOX2 Positive Cells (%)
D
Overall GFAP Intensity in MB Series
Overall GFAP Intensity
MB Series: OTX2 Positive Rate Correlation Analysis
R = 0.92, p = 0.00045
Tumor OTX2 Positive Rate (%)
Organoid OTX2 Positive Rate (%)
MB Series: SOX2 Positive Rate Correlation Analysis
R = 0.73, p = 0.024
Tumor SOX2 Positive Rate (%)
Organoid SOX2 Positive Rate (%)
MB Series: GFAP Intensity Correlation Analysis
R = 0.62 p = 0.077
Tumor GFAP Intensity
Organoid GFAP Intensity

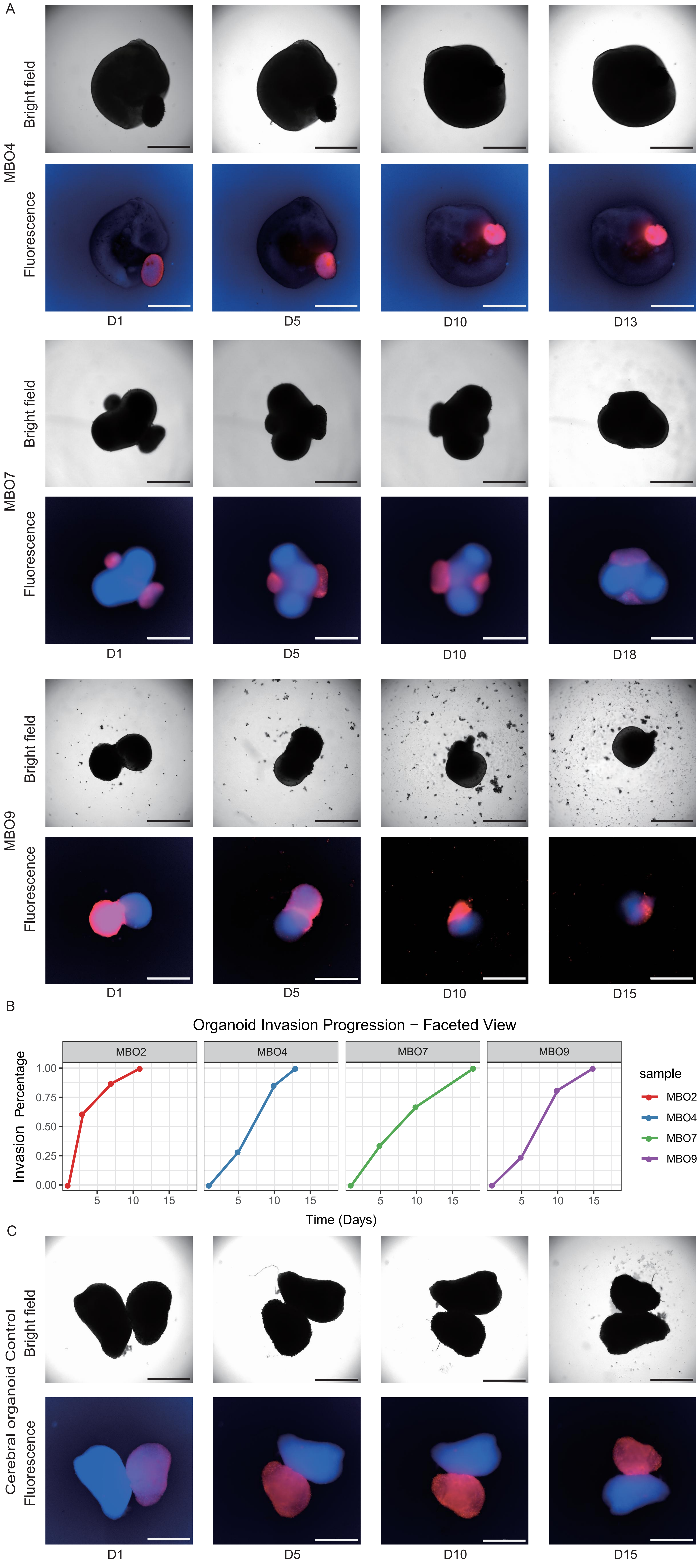

A
MBO4
Bright field
Fluorescence
D1
D5
D10
D13
MBO7
Bright field
Fluorescence
D1
D5
D10
D18
MBO9
Bright field
Fluorescence
D1
D5
D10
D15
B
Organoid Invasion Progression − Faceted View
MBO2
MBO4
MBO7
MBO9
Invasion Percentage
1.00
0.75
0.50
0.25
0.00
5
10
15
Time (Days)
sample
MBO2
MBO4
MBO7
MBO9
C
Cerebral organoid Control
Bright field
Fluorescence
D1
D5
D10
D15

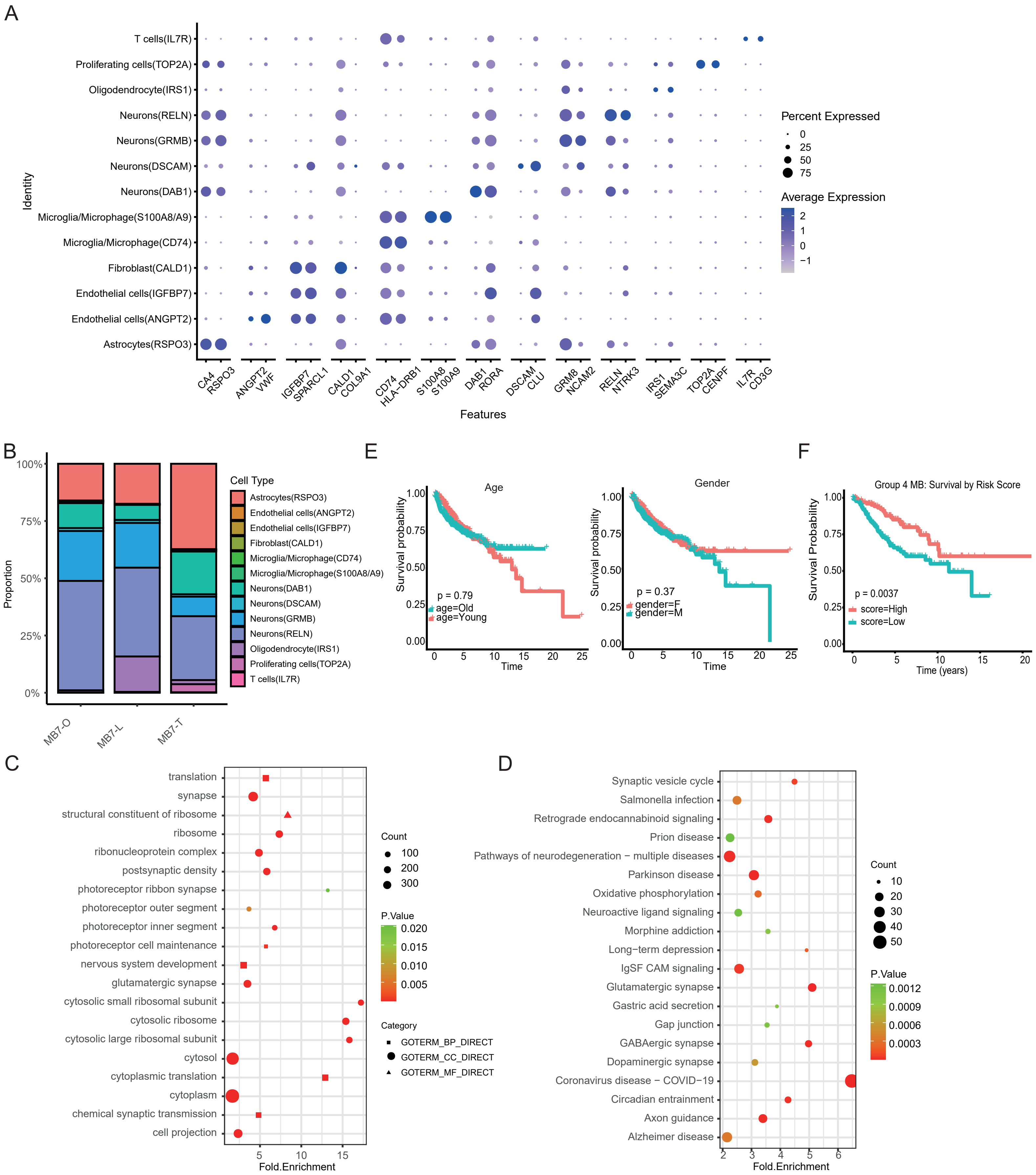
A
T cells(IL7R)
Proliferating cells(TOP2A)
Oligodendrocyte(IRS1)
Neurons(RELN)
Neurons(GRMB)
Neurons(DSCAM)
Neurons(DAB1)
Microglia/Microphage(S100A8/A9)
Microglia/Microphage(CD74)
Fibroblast(CALD1)
Endothelial cells(IGFBP7)
Endothelial cells(ANGPT2)
Astrocytes(RSPO3)
Identity
CA4 RSPO3 ANGPT2 VWF IGFBP7 SPARCL1 CALD1 COL9A1 CD74 HLA−DRB1 S100A8 S100A9 DAB1 RORA DSCAM CLU GRM8 NCAM2 RELN NTRK3 IRS1 SEMA3C TOP2A CENPF IL7R CD3G
Features
Percent Expressed
0
25
50
75
Average Expression
2
1
0
−1
B
Proportion
100%
75%
50%
25%
0%
MB7-O
MB7-L
MB7-T
Cell Type
Astrocytes(RSPO3)
Endothelial cells(ANGPT2)
Endothelial cells(IGFBP7)
Fibroblast(CALD1)
Microglia/Microphage(CD74)
Microglia/Microphage(S100A8/A9)
Neurons(DAB1)
Neurons(DSCAM)
Neurons(GRMB)
Neurons(RELN)
Oligodendrocyte(IRS1)
Proliferating cells(TOP2A)
T cells(IL7R)
E
Age
Survival probability
p = 0.79
age=Old
age=Young
Time
Gender
p = 0.37
gender=F
gender=M
Time
F
Group 4 MB: Survival by Risk Score
Survival Probability
p = 0.0037
score=High
score=Low
Time (years)
C
translation
synapse
structural constituent of ribosome
ribosome
ribonucleoprotein complex
postsynaptic density
photoreceptor ribbon synapse
photoreceptor outer segment
photoreceptor inner segment
photoreceptor cell maintenance
nervous system development
glutamatergic synapse
cytosolic small ribosomal subunit
cytosolic ribosome
cytosolic large ribosomal subunit
cytosol
cytoplasmic translation
cytoplasm
chemical synaptic transmission
cell projection
Fold.Enrichment
Count
100
200
300
P.Value
0.020
0.015
0.010
0.005
Category
GOTERM_BP_DIRECT
GOTERM_CC_DIRECT
GOTERM_MF_DIRECT
D
Synaptic vesicle cycle
Salmonella infection
Retrograde endocannabinoid signaling
Prion disease
Pathways of neurodegeneration − multiple diseases
Parkinson disease
Oxidative phosphorylation
Neuroactive ligand signaling
Morphine addiction
Long−term depression
IgSF CAM signaling
Glutamatergic synapse
Gastric acid secretion
Gap junction
GABAergic synapse
Dopaminergic synapse
Coronavirus disease − COVID−19
Circadian entrainment
Axon guidance
Alzheimer disease
Fold.Enrichment
Count
10
20
30
40
50
P.Value
0.0012
0.0009
0.0006
0.0003

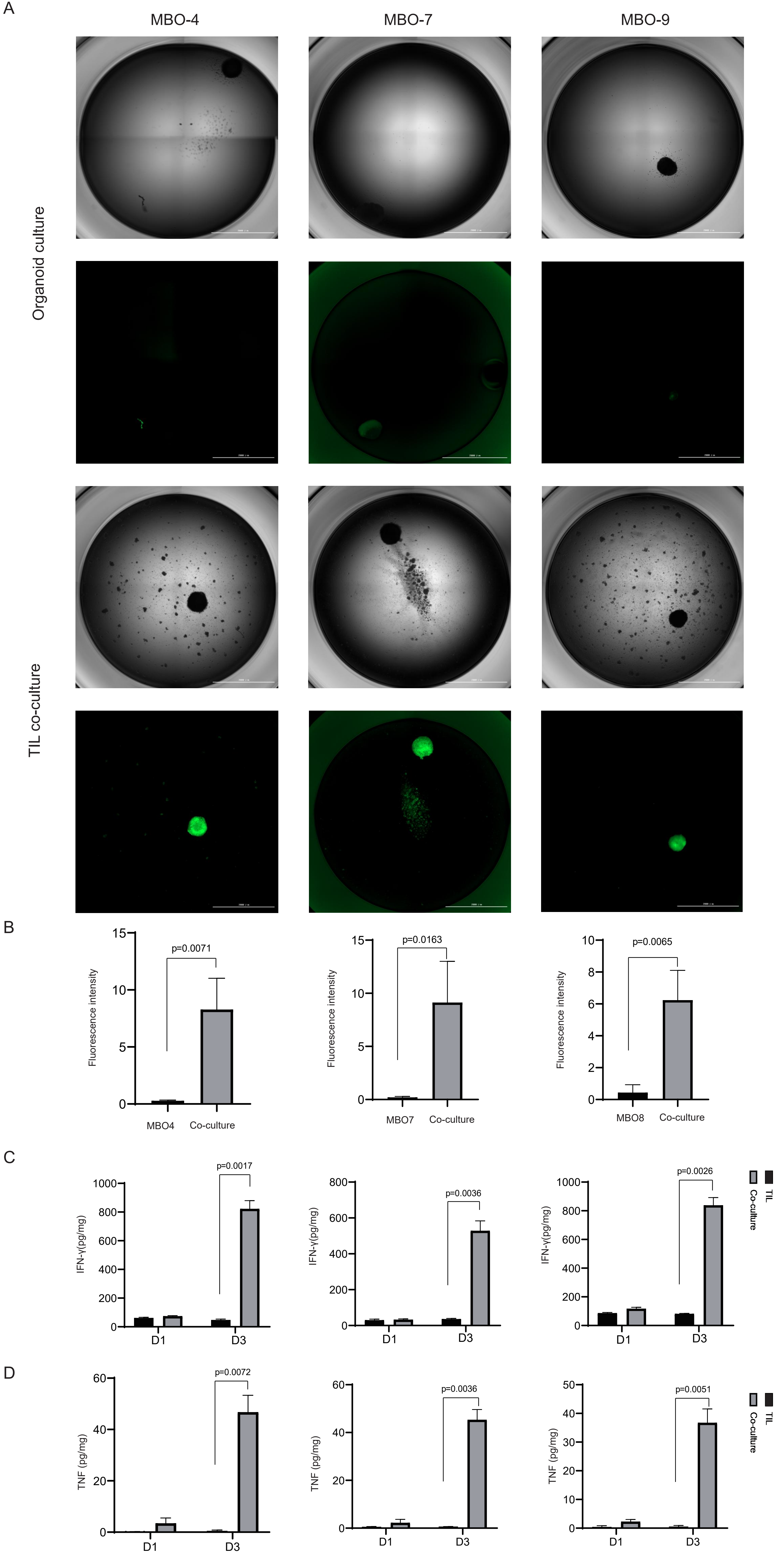

A
MBO-4
MBO-7
MBO-9
Organoid culture
TIL co-culture
B
Fluorescence intensity
p=0.0071
MBO4
Co-culture
p=0.0163
MBO7
Co-culture
p=0.0065
MBO8
Co-culture
C
IFN-γ(pg/mg)
p=0.0017
p=0.0036
p=0.0026
D1
D3
TIL
Co-culture
D
TNF (pg/mg)
p=0.0072
p=0.0036
p=0.0051
D1
D3
TIL
Co-culture

Table S1. Sample information of medulloblastoma

ID	Date	Gender	Age	Subtype
MB1	2023/7/18	female	13	WNT/CTNNB1 mutation
MB2	2023/8/16	male	10	SHH/TP53 wildtype
MB3	2023/8/30	male	7	WNT/CTNNB1 mutation
MB4	2023/12/6	male	7	Group4
MB5	2023/12/19	male	6	Group4
MB6	2023/1/9	female	5	SHH/TP53 mutation
MB7	2023/2/5	female	10	Group4
MB8	2024/4/3	female	9	SHH/TP53 wildtype
MB9	2024/4/8	male	1	SHH/TP53 wildtype
MB10	2024/9/27	male	10	Group4

Table S2. Immunohistochemical Comparison Between 10 MBOs and Corresponding Primary Tumors

Sample	Subtype	Nuclear_Positive_Percentage（%）					Overall_Intensity		
		KI67	OTX2	P53	SOX2	β-Catenin	β-Catenin	GAB1	GFAP
MB1-O	WNT	13.41	1.2	4.55	4.93	80.65	0.1203	0.0159	0.0368
MB1-T	WNT	11.63	2.84	3.87	2.02	67.53	0.0839	0.0041	0.0353
MB2-T	SHH/TP53-	22.05	13.73	38.47	17.55	4.93	0.0381	0.0933	0.0307
MB2-O	SHH/TP53-	22.5	26.47	45.05	37.37	5.49	0.0393	0.1072	0.0225
MB2-R	SHH/TP53-	28.61	21.99	45.91	22.78	3.28	0.0208	0.0832	0.0376
MB2-IC	SHH/TP53-	30.77	28.68	41.64	53.76	5.04	0.0329	0.0691	0.026
MB2-SQ	SHH/TP53-	35.89	16.13	38.72	69.52	8.51	0.0435	0.0794	0.021
MB3-O	WNT	1	4.49	0.16	2.8	42.11	0.0883	0.0024	0.047
MB3-T	WNT	1.9	4.85	0	3.59	57.14	0.1364	3e-04	0.0494
MB4-O	G4	12.88	96.23	1.96	7.9	0	0.0054	0.0049	0.0453
MB4-T	G4	10.46	84.31	0.3	3.17	0	0.0033	0.0014	0.0433
MB5-O	G4	18.32	68.38	0.1	15.75	0.97	0.023	0.0032	0.0256
MB5-T	G4	11.37	47.65	0.2	8.9	3.55	0.0626	0.0114	0.0417
MB6-O	SHH/TP53+	10.91	0	0.46	10.38	10.27	0.0774	0.1183	0.0453
MB6-T	SHH/TP53+	7.57	0.34	0.28	5.4	7.54	0.0546	0.0796	0.0595
MB7-O	G4	20.31	52.43	0	1.63	4.37	0.0366	0.0015	0.0357
MB7-T	G4	10.66	83.51	0.72	1.58	0	7e-04	0.0147	0.0567
MB8-O	SHH/TP53-	2.26	4.46	28.89	1.6	1.07	0.0086	0.0651	0.0343
MB8-T	SHH/TP53-	15.02	5.33	13.58	3.85	1.65	0.0358	0.081	0.032
MB9-O	SHH/TP53-	4.76	7.52	31.18	8.25	9.23	0.0506	0.1031	0.0251
MB9-T	SHH/TP53-	5.94	3	20.53	11.9	7.35	0.0327	0.0541	0.0361
MB10-O	G4	0.39	29.86	0	1.97	5.94	0.0423	0.01	0.049
MB10-T	G4	0.7	33.97	0.33	1.63	6.74	0.0571	0.0477	0.0592
